# Adjuvant Novel Nanocarrier-Based Targeted Therapy for Lung Cancer

**DOI:** 10.3390/molecules29051076

**Published:** 2024-02-29

**Authors:** Kangkan Sarma, Md Habban Akther, Irfan Ahmad, Obaid Afzal, Abdulmalik S. A. Altamimi, Manal A. Alossaimi, Mariusz Jaremko, Abdul-Hamid Emwas, Preety Gautam

**Affiliations:** 1 School of Pharmaceutical and Population Health Informatics (SoPPHI), DIT University, Dehradun 248009, India; sarma_kangkan@outlook.com (K.S.); 1000015383@dit.edu.in (P.G.); 2 Department of Clinical Laboratory Sciences, College of Applied Medical Sciences, King Khalid University, Abha 62521, Saudi Arabia; 3 Department of Pharmaceutical Chemistry, College of Pharmacy, Prince Sattam bin Abdulaziz University, Al-Kharj 11942, Saudi Arabia; o.akram@psau.edu.sa (O.A.); as.altamimi@psau.edu.sa (A.S.A.A.); m.alossaimi@psau.edu.sa (M.A.A.); 4 Smart-Health Initiative (SHI) and Red Sea Research Center (RSRC), Division of Biological and Environmental Sciences and Engineering (BESE), King Abdullah University of Science and Technology (KAUST), Thuwal 23955, Saudi Arabia; mariusz.jaremko@kaust.edu.sa; 5 Core Labs, King Abdullah University of Science and Technology (KAUST), Thuwal 23955, Saudi Arabia; abdelhamid.emwas@kaust.edu.sa

**Keywords:** TME, nanocarrier, lung cancer, vascular modification, PTT, ROS, targeted drug delivery, liposome, metallic nanoparticle

## Abstract

Lung cancer has the lowest survival rate due to its late-stage diagnosis, poor prognosis, and intra-tumoral heterogeneity. These factors decrease the effectiveness of treatment. They release chemokines and cytokines from the tumor microenvironment (TME). To improve the effectiveness of treatment, researchers emphasize personalized adjuvant therapies along with conventional ones. Targeted chemotherapeutic drug delivery systems and specific pathway-blocking agents using nanocarriers are a few of them. This study explored the nanocarrier roles and strategies to improve the treatment profile’s effectiveness by striving for TME. A biofunctionalized nanocarrier stimulates biosystem interaction, cellular uptake, immune system escape, and vascular changes for penetration into the TME. Inorganic metal compounds scavenge reactive oxygen species (ROS) through their photothermal effect. Stroma, hypoxia, pH, and immunity-modulating agents conjugated or modified nanocarriers co-administered with pathway-blocking or condition-modulating agents can regulate extracellular matrix (ECM), Cancer-associated fibroblasts (CAF),Tyro3, Axl, and Mertk receptors (TAM) regulation, regulatory T-cell (Treg) inhibition, and myeloid-derived suppressor cells (MDSC) inhibition. Again, biomimetic conjugation or the surface modification of nanocarriers using ligands can enhance active targeting efficacy by bypassing the TME. A carrier system with biofunctionalized inorganic metal compounds and organic compound complex-loaded drugs is convenient for NSCLC-targeted therapy.

## 1. Introduction

According to the World Health Organization (WHO), lung cancer was the second-highest diagnosis (11.4%) and the leading cause of death (18%) among all forms of cancer in 2020 [1]. The 5-year survival rate of lung cancer patients worldwide was 19% from 2010–2014. A few countries, such as Japan (33%), Israel (27%), and the Republic of Korea (25%), had a higher survival rate [2]. The prime reasons for the low survival rate are late-stage diagnosis, lack of awareness, socioeconomic conditions, environmental contamination, and the metastatic and intra-tumoral heterogeneity nature of the tumor [3,4,5,6].

The common etiological factors for lung cancer development are tobacco smoking (which causes 80% of cases in the United States and other countries), occupational asbestos exposure (5–10% globally), cannabis or marijuana smoking (4% in the USA in 2002–2014), radon exposure (10% in the Western World), air pollution, group 1 carcinogen arsenic exposure, inflammation and cellular damage during respiratory infection, chronic obstructive pulmonary disease-related inflammation and scarring, and family history of lung cancer [7,8,9,10,11,12,13,14,15]. Other associated increased risk factors for lung cancer are systemic sclerosis patients, smoker breast cancer survivors, HIV-infected patients with idiopathic pulmonary fibrosis, certain fibrotic pneumoconioses patients, and lung cancer survivors.

The etiological agents such as radon, smoke, and cannabis have free radicals, reactive oxygen species, and reactive electrophiles [16,17,18]. Depending on the dose, dimension, durability, and surface reactivity that react with nitrogen and oxygen atom lesions in the DNA, modifying nucleotides to distort the base pairing leads to incorrect nucleotide incorporation during replication [19,20,21,22,23,24]. Cell repair mechanisms can repair DNA damage. But the escape portions change the coding of the DNA. Repeated exposure to etiological factors leads to a series of genomic changes such as copy number variations (CNVs), single-nucleotide variations (SNVs), and insertions/deletions (INDELs) of exomes in the autosomal chromosome that lead to a permanent change in the sequence and that start from the primary tumor, followed by metastasis via circulating tumor cells [25,26,27]. Genetic mutations affect protein synthesis, disrupt cell cycle progression, and promote carcinogenesis. Circulatory tumor cells for metastatic cancer and the genomics of the tumor cells for the non-invasive type help in diagnosis and prognosis. Circulating tumor cell analysis is helpful for the prediction of disease progression, the survivability of patients, and personalized therapy because cell-free DNA fragments are found in peripheral blood [24,25,26,27,28,29,30]. Lung cancer occurs through either one or a combination of the etiological factors that affect and cause mutation of protooncogenes, tumor suppressor genes, DNA repair gene dysfunction, apoptotic mechanism erosion, limitless telomere replication, sustained angiogenesis, increment of invasion, metastasis, and escape from immunity [30,31,32,33,34,35].

Histologically, lung cancer is classified into non-small cell lung cancer (NSCLC, 85%), and small cell lung cancer (SCLC, 13%) [36]. Further, NSCLC subdivides into lung adenocarcinoma (40%), squamous cell carcinoma (25–30%), and large cell carcinoma (5–10%) [34,35,37]. In 2015, the WHO modified the classification of lung cancer based on immunohistochemistry, genetic studies for personalized treatment strategies, and small biopsy and cytologic samples [36,38,39]. This new classification objective is to overcome drug resistance, intracellular accumulation, metastasis, invasion, side effects, and toxicity, and develop a more personalized novel treatment regime [40]. The current treatment regime depends upon the stage of cancer progression, the health of the patients, and affordability at the time of diagnosis. The different treatment methods are surgery (wedge resection, segmental resection, lobectomy, and pneumonectomy), radiation therapy, chemotherapy, stereotactic body radiotherapy, targeted drug therapy, immunotherapy, and palliative care.

Surgery remains the top priority for multimodality cancer patients with advanced stages (III and IV). It is better suited for stage I and II lung cancer because surgery eliminates it before it has spread to lymph nodes or distant sites. Neo-adjuvant therapy is commonly used before surgery to reduce tumor size. The common side effects of lung cancer surgery are severe chest pain, blood clots, bronchopleural fistula, excessive bleeding, collapsed lungs, depression, difficulty breathing, fatigue, loss of appetite, gastrointestinal problems, heart problems, and a sore mouth [41,42,43].

Radiation therapy acts as both a primary and adjuvant therapy for cancer patients by damaging the DNA of the cancerous cell in a dose-dependent manner. It can generate antitumor T cells by activating the stimulator of the interferon genes (STING) pathway to produce interferon-1. Interferon-1 can deliver the DNA fragment to dendritic cells to produce antitumor T cells. Radiation therapy also helps in the trafficking of chemokines by homing T cells to the TME and modulating immunity [44,45,46]. The adverse effects of radiation therapies include damage to epithelial surfaces, intestinal discomfort, inflammation, infertility, fibrosis, epilation, lymphedema, polyneuropathy, and dryness [47].

Chemotherapy is another prominent therapy to control the growth of cancer cells. It can be used before and after surgery in NSCLC patients and with targeted or radiation therapy in the late stage of cancer. Excessive toxicity makes it controversial regarding the effective use of chemotherapeutic agents in lung cancer treatment. Chemotherapeutic agents can damage the DNA or RNA of cancer cells to inhibit their reproduction. The common adverse effects of chemotherapy are nausea, vomiting, sore mouth, weight change, and hair loss [48,49].

Stereotactic body radiotherapy (SBRT) is a neo-adjuvant or adjuvant therapy for operable patients with co-existing interstitial lung disease. It is an alternative therapy for non-operable early-stage localized NSCLC patients. Using target delineation, motion management, image guidance, and dose optimization in treatment planning is possible with this technique. The reported toxicity level for this treatment regime is lower. The adverse effects of SBRT are shortness of breath, chest wall pain, urinary irritation, nausea, and vomiting [50,51,52].

Targeting therapy is designed to alter the specific abnormalities in the cancer cells and their microenvironment. This therapy acts as an adjuvant in the early as well as late stages of the disease’s progression. It involves targeting specific genes or proteins using a drug-loaded carrier system to deliver them to a projected site. A modification of the carrier system enhances the efficacy of the drug at the targeted site. The limitations of conventional therapy can be overcome by using targeted drug delivery systems. It may cause site-specific nano-toxicity and minimal toxicity to surrounding cells. Optimization of targeted drug delivery is one of the biggest challenges [40,53,54,55,56].

Immunotherapy is a treatment regime to identify optimal neoantigen candidates and inhibit the functions of tumor cells. It produces higher effectivity when tumor-neoantigen reaches the dendritic cell and is recognized by the T-cell receptor. Then, it circulates through the periphery, overcomes the immunosuppression of the tumor cell, causes stimulation within the tumor cell, and finally releases the potent molecule to kill the tumor cells. This treatment regime can be used as adjuvant therapy after surgery with chemotherapy, radiation, hormones, and targeted therapy. Fatigue, cough, nausea, itching, skin rash, constipation, diarrhea, and joint pain are a few common adverse effects of immunotherapy [57,58].

Palliative care is an adjuvant therapy to enhance the quality of life for patients with severe diseases such as lung cancer. It focuses on the physical, psychological, spiritual, and practical burdens after a disease diagnosis. It continues with surgery, radiotherapy, and immunotherapy [59].

Though the advancement of the treatment regime impacted the treatment profile, late-stage diagnosis (metastasis stage) creates a burden [3,4,5]. So, the emphasis has increased on chemotherapy and pathway-blocking agents through targeted drug delivery systems for advanced-stage patients [60].

Nanocarriers, a colloidal preparation with a higher number of pores, can be used for the diagnosis and delivery of targeted drugs, nucleic acids, proteins, and diagnostic agents at the desired rate and time to the targeted site through passive, active targeting, pH, and temperature specificity to block pathways and reduce systemic drug toxicity [61,62,63]. As a result of NPs’ small size, tailored surfaces, improved solubility, and multifunctionality, NPs provide superior stability, solubility, and bioavailability. It delivers the magnetic, thermal, electrical, and optical forms of active pharmaceutical ingredients used as targeted radiational, chemotherapeutic, gene therapeutic, immunotherapeutic, and combinational agents to treatment sites through the EPR effects. Depending upon the types, nature, and intention of the use of drugs, they are encapsulated, entrapped, dissolved, or absorbed in nanocarriers. Nanocarriers, a circulating cargo, can enhance the circulation lifetime, permeability, and retention of active pharmaceutical ingredients [64,65]. Viral vector nanocarriers can deliver nucleic acid therapies [66]. In short, a nanocarrier is a system that can control, manipulate, and fabricate micron-sized structures and devices.

Optimization of the physical properties of NPs facilitates the delivery of drugs at a specific rate and time to the desired sites. In addition to protecting the active medicament from premature degradation, nanocarriers control and improve drug distribution through intracellular accumulation, penetration, and shelf life [67,68]. Nanobiocarriers are bioactive or targeting vectors or ligands that deliver the active pharmaceutical moiety to mimic and control unnecessary cellular extravasations, growth, and cellular events. Additionally, nanobiocarriers enable the delivery of drugs with optimal biocompatibility, biointeraction, safety, and efficacy [69].

## 2. Drug Delivery Constraints in Lung Cancer Management

The self-defense mechanism of the respiratory tract impacts drug delivery and absorption on the lung surface through mechanical, chemical, and immunological barriers [69]. A pulmonary drug delivery method can overcome biological barriers by avoiding the cough reflex, using aerosol, interacting with airway surface liquid and mucus to reach cellular targets, overcoming systemic absorption, degradation, or clearance of the active molecule to reach intracellular targets, and maintaining drug concentrations within therapeutic windows until the next administration [70].

However, in lung cancer, the biological barriers act differently, and tumor heterogeneity also adds instruments [69]. Different attempts have been made to solve these problems. The control of tumor endothelia and sprouting angiogenesis are two such attempts. Generally, sprouting angiogenesis is caused by the vascular endothelial growth factor’s (VEGF) action on its receptor. So, selecting a drug that can access VEGF can enhance the vascular permeability of chemotherapeutics [71]. Another method is to control vascular co-option. Vascular co-option is a process of tumor cell interaction and exploitation with normal tissue vasculature by migrating through the host tissue’s blood vessels in a VEGF non-dependent manner. Clinical tumor-coopted vessels can be identified through specific functional biomarkers or molecular markers [71]. The other ways to overcome the biological barriers are to control vasculogenic mimicry, intussusceptions, and vasculogenesis. Using high intestinal fluid pressure in the tumor neovasculature may be another approach to overcome the barriers because negative or low transcapillary pressure gradients of normal tissue enable outer flow into its tissues. By passing the heterogenic biological barriers of cancer, paracellular transport across the endothelium can transport chemotherapeutic fluids by maintaining concentration gradients. Furthermore, transcellular routes allow molecules to travel through endothelial cells and are an alternative strategy for cancer drug delivery that needs to be considered by current therapeutics. Again, normalization of vessels, vascular promotion, and tumor-specific peptide incorporation with nanocarriers can improve passive drug delivery into tumors. Finally, active transvascular delivery using caveolae pumping may be another way to overcome biological barriers [71].

Targeted therapy is the formulation approach to overcome the lung surface barriers to the targeted site. The lung-targeted therapy enhances the safety and efficacy of the drugs. Examples include topical targeted lung therapy, which acts rapidly in low doses with no systemic side effects. A systemic targeted lung therapy bypasses the gastrointestinal tract and has a better pharmacokinetic profile [69]. The relationship between the active drug’s physicochemical properties and its biological functions affects the development of targeted therapy and treatment profiles [72]. The particle size distribution of active pharmaceutical ingredients, or nanocarriers, is an example. Particles of larger size deposit more in the lung’s central airways, whereas fine particles deposit more in the peripheral airways [73]. The geometrical shape of the nanocarriers affects drug delivery to the neovasculature. Geometrical shapes influence dissolution/diffusion rates and the release of drugs [74]. It also impacts the circulation, margination, adhesion, and internalization of the nanocarriers and active pharmaceutical ingredients. Generally, cylindrical and spherical dispersed drugs have a lower dissolution/diffusion ratio, and the solubility/drug loading ratio is lower, which enhances drug release. A drug’s release pattern may vary based on its geometric shape. Again, surface charges are the distribution of the drug carriers. In addition to surface charges and geometrical shapes, surface modifications can improve drug delivery effectiveness in nanocarriers [52,53]. A modified cuboidal cyclodextrin metal–organic framework is one example of delivering targeted drugs to injured blood vessels [75]. In addition, comparing the ratio between the metalloproteinase and its inhibitor in the targeted site and consequently choosing the type of NP drug delivery system can enhance the vascularization of the NPs [76,77,78]. The development of the targeted drug delivery system after the physicochemical properties are modulated according to biological functions can optimize the delivery of the active drug to the lung cancer site.

The mononuclear phagocytes of the immune system reduce the reach of nanotherapeutics through opsonization and sequestration processes. It occurs in a protein corona around NPs using the opsonization and sequestration processes. The formation of protein corona depends upon the size and surface chemistry of the NPs. After protein corona formation, it absorbs the NPs, internalizes them, fuses them to the lysosomes, and reduces their specificity [65,78,79,80,81,82,83].

In this study of nanocarrier-based targeting drug delivery to overcome the TME barriers, we have found that particle size and active targeting using receptor-based bioconjugating agents play roles in bypassing TME to enhance the targeting efficacy of the loaded drug. Different studies on lung cancer have found that inflammatory mediators are overexpressed, especially IL-6 [84]. A high-affinity protein can block it. So, nanocarriers biofunctionalized with proteins such as RGD can be useful for targeting cancer cells, and the results of a few studies also complement them [85]. Another study found that folic acid deficiency promotes IL-6/JAK-1/pSTAT3 interactions in astrocytes after ischemia-reperfusion. So, folic acid-biofunctionalized nanocarriers may be another approach to improving targeting precision [86]. Further, the PEGylation of NPs reduces the interaction with serum proteins and enhances the stability of the nanocarrier in the reticuloendothelial system [87].

Functionalizing the NPs according to the targeted profile through the intravenous route can decrease the circulation’s lifetime. Using self-peptide, biomimetic particle coating, and conformation-changing coating molecules on the surface of the NPs can overcome this biological barrier [65,88,89,90]. 

Cancer cells chisel their TME using different factors, such as the release of chemokines and cytokines. These secretions reprogrammed the environment for further tumor growth and disease progression. NPs can passively and limitedly reach the TME through the EPR effect. The tumor heterogeneity acts as a barrier for drug delivery to the TME through uncontrolled vascular events, resistance produced by the stroma, hypoxia, pH, and immune reshaping. For stable drug delivery to the targeted TME, there is a need for favorable vascular network events, regulation of stromal activities, or manipulation of hypoxia, pH, and immunity. In a heterogenic TME, the incremental demand for nutrients increases growth factors and forms leaky vessels. It increases the angiogenesis of tumor cells. It also enhances the interstitial fluid pressure through the leaky vessels and decreases blood flow to the site. So, the drug-loaded NPs cannot reach and accumulate in the targeted space [91,92,93,94,95,96,97,98,99,100,101]. In lung cancer treatment, multiple drug resistances decrease the effectiveness of the treatment regime. A combination of medications for respiratory tract disease changes the compliance rate of the drugs. The modulation of the TME using a single drug therapy with multiple targeting strategies can overcome these issues [92,102,103,104]. A few strategies to optimize drug delivery to the TME are active targeting, TME modulation, and TME-responsive targeted drug delivery [94,101,105].

Nanocarriers also have potential risks and downsides. Nanocarriers can trigger an immune response, leading to inflammation or hypersensitivity reactions. This immune response can be more pronounced if the nanocarriers are derived from foreign materials or have surface properties. Mitigation strategies include using biocompatible and biodegradable materials for nanocarriers or modifying the surface of the nanocarriers to minimize immune recognition [106]. Nanocarriers are designed for targeted delivery but can accumulate in non-target tissues or organs. This can result in off-target effects and potential toxicity. Preclinical studies and careful formulation design can help minimize this risk by optimizing the specificity and stability of the nanocarriers [107]. Nanocarriers may exhibit inherent toxicity if not adequately eliminated from the body. Rigorous toxicity evaluations and optimization of nanocarrier properties, such as size, surface charge, and composition, can help mitigate this risk. Nanocarriers can also experience drug leakage or premature release of the therapeutic payload before reaching the target site. This can result in suboptimal drug concentrations at the intended site and reduce its efficacy. Strategies such as improved encapsulation techniques, surface modifications, or utilizing stimuli-responsive nanocarriers can help minimize premature drug release. Targeted therapy using nanocarriers can be affected by drug resistance mechanisms and the heterogeneity of lung cancer tumors. Combining nanocarrier-based therapy with other treatment modalities or developing strategies to address drug resistance can help overcome this limitation [106,107].

## 3. Nucleic Acid Role in Lung Cancer Management

Cancer is an acquired disease of genetic alteration. Nucleic acids have a promising treatment profile for cancer. This genetic alteration can be improved using the delivery of DNA and other nucleic acids to control the genetic expression profile of target cells. The delivery of nucleic acid to the targeted cell is challenging due to its instability, off-target effects, and traversal of biological barriers [108].The delivery of nucleic acids to the targeted site can be achieved using a nucleic acid cargo or nanocarrier as the nucleic acid vehicle. A nanocarrier charge can deliver DNA or mRNA to overexpress a gene, small interfering RNAs or microRNAs to knock down a gene, or nucleic acids to trigger pattern-recognition receptors to stimulate the immune system [109]. A plasmid containing both a promoter and the gene of interest is used to treat DNA overexpression by bypassing the plasma membrane and nuclear envelope. After reaching the nucleus, it exports and transcribes into mRNA, which is translated into the desired protein in the cytoplasm. Single-stranded mRNA can also be used for the same purpose, but it is less stable and has a lesser chance of undesired insertion into the genome, such as plasmid DNA, to cause mutagenesis [108,109,110]. However, RNAi can interrupt mRNA translation to decrease target gene expression, and this problem can be solved using short-length dsRNA such as siRNA. Although the sequence of nucleic acids can have functional impacts on biological targets, many physical and chemical considerations are not heavily dependent on the nucleic acid sequence encapsulated in a nanocarrier for delivery. So, the chemical and physical properties of the nucleic acid should be considered [108,109,110]. 

Co-delivery of multiple nucleic acids of the same type but with different sequences in a single delivery vehicle follows the same design principles, necessitating changes to nanocarrier design to deliver distinct cellular and subcellular locations. Again, tumor heterogeneity and MDR cause multiple therapeutic agents to target different cellular pathways. However, the multitargeted nucleic acid cargo can cause intrinsic toxicity and virus immunogenicity to prevent repetitive administration [108,109]. The challenges of nucleic acid cargo are the physical and chemical properties’ similarity and the overlap of extracellular and intracellular trafficking routes. As nucleic acids possess a negative charge in their structure, generally positively charged polymers can be used to prepare NPs. Cationic polymers such as poly(l-lysine), polyethyleneimine, polyamidoamine, poly(beta-amino ester), and cationic lipids are used [109]. Again, the size and physical properties of the nucleic acid impact its loading on nanocarriers. Further, surface modification of the nucleic acid NP improves its cellular uptake at the targeted site. Commonly used nanocarriers for nucleic acid delivery are liposomes, SLN, polymeric, gold, mesoporous silica, and iron oxide NPs [111].

## 4. Strategies to Overcome the Tumor Microenvironment

### 4.1. Vascular Remodulation

**Figure 1:**
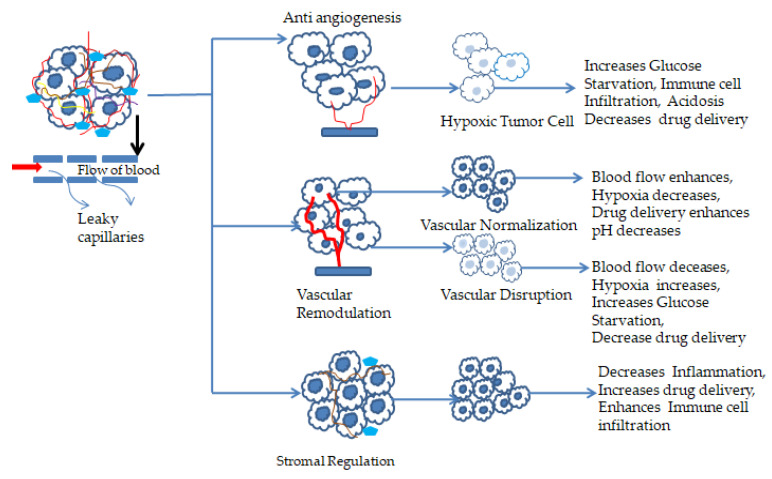
Modulation of tumor blood vessels to enhance cancer therapy.

**Figure 2:**
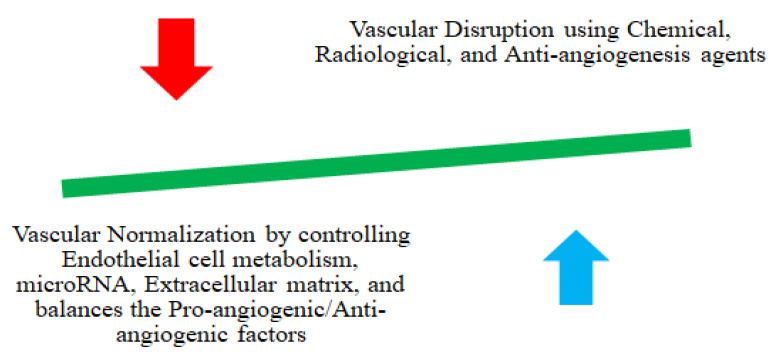
Vascular remodulation.

The vascular network events can be modified using either disrupting agents or normalizing them (Figure 1 and Figure 2). Disrupting vascular events differs from anti-angiogenesis strategies, and when used with chemo, radio, and angiogenesis-inhibiting therapeutic agents, it enhances treatment efficacy (Figure 1). Recently, researchers reported that the co-administration of combretastatin A4 (CA4) NPs with doxorubicin, CA4 NPs with imiquimod, and nanocomposite hydrogel antitumor therapy with near-infrared radiation enhances the treatment efficacy [105,112,113,114,115,116]. Vascular normalization is another approach to re-modulate vascular events that enhances the effectiveness of chemotherapy, radiation therapy, and immunotherapy by reducing tumor invasion and metastasis (Table 1). In addition, the normalization process targets endothelial cell metabolism, microRNA, and extracellular matrix and balances proangiogenic and antiangiogenic factors [117,118]. Anti-VEGF-receptor-2 antibody DC101 modulates NPs, and nitric oxide deliveries with nanocarriers are a few reported approaches that cannormalize vascular events [112,118,119,120,121].

### 4.2. Stromal Regulation

**Figure 3:**
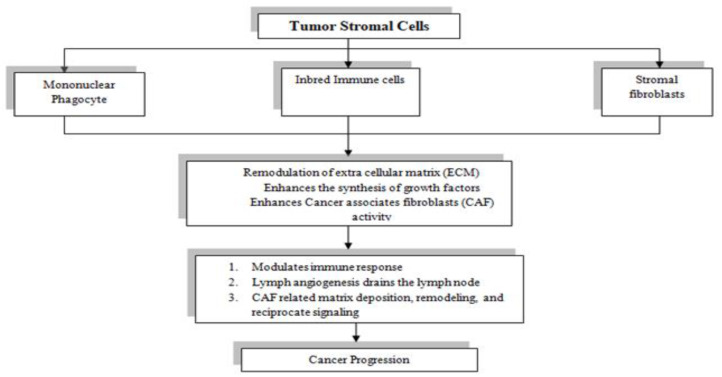
Tumor progression through stromal regulation.

In cancer, stromal cells lose their tumor-suppressing abilities and promote tumor growth, invasion, and metastasis. Stroma regulates ECM synthesis, degradation, and signaling pathways (Figure 3) [122]. So, different attempts have been made to enhance stromal regulation.

Researchers reported that metelimumab (a transforming growth factor-β ligand-blocking antibody) conjugate NPs and prolyl-4-hydroxylase inhibitor (which inhibits collagen synthesis in vascular smooth muscle cells) conjugate NPs enhance drug effectivity through ECM synthesis through stromal regulation [123,124,125,126,127,128]. Further research has indicated that volociximab inhibits angiogenesis by preventing integrins from binding to fibronectin (Table 1). Volociximab can be more effective when combined with other tumor-mimicking drugs based on its ability to modify ECM signaling [123,129]. Evidence suggests hyaluronidase, collagenase, and putrescine inhibition can destroy the ECM [122,130,131]. The preparation of an artificial extracellular matrix (AECM) based on laminin (LN)-mimic peptide hydrogel-fabricated NPs loaded with a drug can enhance effectivity and mimic the ECM [92,132,133]. Another factor that changes the stroma at the invasion front is carcinoma-associated fibroblasts (CAFs). This problem can be solved by either directly disrupting or reducing CAF activity. Applying the disruption strategy, researchers reported that artemisinin (which inhibits vimentin expression) as a capping agent for an NP loaded with therapeutics treats the invasion process. In general, vimentin expression increases the migration and invasion of cancer cells. Further, fibroblast activation protein was overexpressed in the stroma. *N*-(4-quinolinoyl)-Gly-(2-cyanopyrrolidine) capping NPs (which inhibit FAP overexpression) with the active drug may regulate stroma [134,135,136,137,138]. Reprogramming the CAFs can delay the disease’s progression. CAFs act as either immune suppressive or supportive agents (Table 1). Angiogenic receptor blockers (ARBs) latently inhibit CAF activity, and ARB nanoconjugates can enhance immune-supportive activity [139,140].

### 4.3. Hypoxia Manipulation

Hypoxia is the normal state in the TME that changes the metabolic pathways. It activates hypoxia-induced factors to regulate energy demand through anaerobic glycolysis. Anaerobic glycolysis increases lactate production, and H+ ions lead to acidosis in the TME [92,96,100,101]. It decreases drug compliance and increases resistance and angiogenesis. Hypoxia can be manipulated through an elevation or decrease in oxygen consumption or by using hypoxia-activated prodrugs [92]. According to the literature, oxygen supply elevates with theranostic upconversion nanoprobe MnO_2_ NPs. The tumor cell produces excessive amounts of H_2_O_2_ and lactic acid. Theranostic MnO_2_ reacts with acidic H_2_O_2_ to produce Mn^2+^ and enhance O_2_ production [141,142]. Encapsulating photothermal therapy with electron-transporting chain-inhibiting agents using NPs reduces oxygen consumption [140]. Further, hypoxia-activated prodrug (HAP) activates spontaneous electron oxidoreductases (Table 1). HAP agents combined with targeted therapy and checkpoint blockers increase the influx into the hypoxic zone [143,144,145].

### 4.4. pH Manipulation

A variation in the external to internal pH of tumor cells causes disturbances in biological functions, such as proliferation, migration, and aggression. pH can be manipulated using small-molecule drugs, acidity-neutralizing inhibitors, or pH-regulating enzymes [146]. One reported mechanism for pH manipulation is acid neutralization using sodium potassium citrate, which increases the blood HCO^3−^ level in the oral dose and neutralizes the TME pH [147,148]. Proton pump inhibitors and carbonic anhydrase IX/XII can be used to inhibit the enzymes that manipulate pH [149,150]. In a study, researchers found that pH-dependent dendritic polyglycerol-co-polycaprolactone-derived polymer NPs loaded with gemcitabine are stable at pH 7.4 and can release the drug in a time-dependent manner with improved cellular uptake in the desmoplastic stroma of pancreatic cancer [151].

### 4.5. Immunity Modulation

Tumor-associated macrophages and regulatory T cells of the TME suppress and escape the immune system. As the TME hinders the trafficking of CD8+ cells, the immune system cannot inhibit the tumor. They decrease nano-therapeutic compliance and increase nano-therapeutic resistance [92,102,103,104,105]. Regulatory T-cell (Treg) inhibition, Tyro3, Axl, and Mertk receptor (TAM) regulation, and myeloid-derived suppressor cells (MDSC) inhibition are a few strategies to overcome immunity. TAM overexpression increases cell survival and decreases apoptotic signaling. Again, TAM down-streaming promotes metastasis via migration and invasion. Polarization of macrophage M1 to M2 by TAM has an immunosuppressive effect. M2 releases immune-suppressive cytokines. M2 blocking agents and M1 reprogramming agents can regulate this immunity suppression. Small-molecule tyrosine inhibitors and TAM receptor-targeted ligands can regulate it [152,153,154,155]. Treg cells regulate T-cell immune responses to maintain cell homeostasis. Adversely, in TME, Treg cells decrease the entry of T cells. Transforming growth factor-β (TGF-β) inhibitors and anti-PD-L1 antibodies reduce the TGF-β signal to promote T-cell infiltration into the TME (Table 1). The cytotoxic T-lymphocyte-associated protein-4 (CTLA-4) antibodies remove the Treg cells and can enhance T-cells’ functions [156]. MDSC induces immune suppression by inhibiting T-cell, NK-cell, and macrophage functions. Targeting and inhibiting phosphatidylinositol 3-kinase (PI3K)δ, PD-L1, CTLA-4, and multi-kinase MDSC can control the same [157,158]. According to a clinical update by the US Patent Office, researchers have found that anti-CTLA-4 antibodies antagonize CTLA-4 to control T-cell functions for controlling immunity [159]. Recently reported research has found that a small-regularity self-replicating RNA intradermal vaccine against SARS-CoV-2 elicits predominantly cellular immunity because of T-cell induction [160].In another study, researchers found that pembrolizumab, as a monotherapy, enhances disease regression with minimal toxicity. Moreover, no researcher has reported a targeted drug delivery system for immunotherapy until now [161,162]. 

### 4.6. Active Targeting

Surface and biomimetic modification of NPs improves the active targeting strategy. As a form of active or ligand-mediated targeting, NPs ligation affinity on their surface ensures that they are retained and taken up by the targeted cells. Ligands (antibodies, proteins, peptides, nucleic acids, sugars, and small molecules such as vitamins) are selected to target surface molecules or receptors overexpressed in diseased organs, tissues, cells, or subcellular domains to benefit from active targeting. Activelytargeted materials must be specific to their targets. This approach aims to enhance NP–cell interactions and drug internalization without affecting overall biodistribution ortoxicity to normal cells [163]. These ligands can recognize and bind to specific receptors or markers on the target cells, facilitating selective uptake and enhancing the targeting efficacy of the NPs along with their specificity, stability, and interaction with target cells or tissues [163]. This strategy bypasses TME and synergizes both active and passive targeting. Cancer cell membrane-coated NPs can carry antigens and drugs to the target [164,165]. Protein-conjugated NPs can leverage both active targeting (targeting ligand-mediated) and passive targeting (based on NP properties, such as size and surface charge) mechanisms. This synergy can enhance targeting efficacy and improve therapeutic outcomes [163,164,165]. Depending onthe choice of protein, conjugation method, and NP characteristics, the targeting of protein-conjugated NPs can be affected. The selection of appropriate proteins and optimization of conjugation strategies should be based on understanding the target cells, tissues, and disease context. Researchers found that folate discs enhanced permeability and photothermal efficacy. So, surface modification with folate can be a better therapeutic approach [153,166]. Recently, researchers have found that folic acid-conjugated chitosan NPs loaded with 5-fluorouracilshow higher cytotoxicity than chitosan 5-fluorouraciland enhance the targeting of tumor cells [167]. Research and development of protein-conjugated NPs are ongoing, aiming to improve their targeting efficacy, therapeutic potential, and clinical translation. In lung cancer patients, researchers have found that protein-functionalized lipid hybrid NPs in response to transferrin exhibit improved therapeutic efficacy when loaded with cis-platin and docetaxel [168]. In another study, researchers reported that cyclic RGD-functionalized cyclodextrin metal–organic NPs loaded with doxorubicin enhanced the drug’s efficacy by 4–5 times. As a result, it exhibits transferrin-dependent targeting that reduces off-targeting [85]. One recent study reported that PLGA NPs functionalized with RGD control the loaded drug delivery and enhance its efficacy [169].

### 4.7. Tumor Environment Responsive Drug Delivery

The stimuli of tumor tissue differ physically and biochemically from those in normal tissues. To improve the effectiveness of the drug delivery to the TME, the nanocarrier must penetrate or should have improved cellular uptake or enhanced drug release at cancer sites [92,170]. Newly designed stimuli-responsive drug delivery systems can overcome the barrier of TME and deliver the required amount of drug to the targeted sites. One such method is a supramolecular architecture based on peptides. In response to the TME, supramolecular architectures based on peptides can convert structurally and allow therapeutics for controlled release. This dissertation emphatically introduces peptide assemblies with a stimulus-responsive structural conversion to acids, high temperatures, and high oxidative potentials in tumor tissues. Functional moieties that respond to cellular stimuli such aspH, glutathione, adenosine triphosphate, reactive oxygen species, enzymes, and inflammatory factors can act as targeting strategies [171,172,173]. Further, the dense ECM prevents larger NPs (>100 nm) from penetrating the tumor after they have extravagated from vessels. After blood circulation, particles with a relatively large size can shrink in size due to internal stimuli such as enzymes, acidic pH, and hypoxia. Using peptides or favorable ligands, NPs entrap and form corona at the TME. This strategy helps to internalize the entrapped drug by interacting with the moiety and reducing its size by engulfing it with TME [170,174,175,176,177,178,179]. 

The strategy to enhance cellular uptake can be the conversion of charge or the detachment of the NP shell. It eliminates long circulation times and cellular uptake by modifying its surface charge. TME cues such as redox potential, acidic pH, and overexpressed enzymes can stimulate the responsive bonds on the surface of NPs to achieve charge reversal (Table 1). A few examples are pH-sensitive PEG coatings and enzyme-sensitive nanovectors [180]. As nanovectors accumulate at tumor sites via the EPR effect, overexpressed MMP-9 can detach the PEG corona to expose RGD and facilitate cellular internalization. The PEG coating cleaves with the TME and removes the PEG shell to enhance cellular uptake [92]. 

Another strategy is that on-demand drug release may result from a hydrophilic–hydrophobic switch triggered by TME signals (Table 1). As a result of the protonation and de-protonation polymers present in the NPs, they can switch from hydrophilic to hydrophobic and trigger drug release at the targeted sites. Cleavage with a sensitive linker to TME can trigger the rapid release of an entrapped drug from a nanocarrier [181,182].

Here is a brief overview of strategies to overcome drug delivery challenges in tumor microenvironments (Table 1).

**Table 1:** Strategy, process, and mechanism with a few examples to overcome the barrier of TME.

Strategies	Process	Mechanism	Example	Ref.
Modulation of TME	Vascular network remodulation	Vascular network disruption and decompression	Co-administration of combretastatin A4 (CA4) NPs with doxorubicin, CA4 NPs with Imiquimod, nanocomposite hydrogel antitumor therapy, and near-infrared radiation	[112,113,114,115,116]
		Normalizing the vascular network	Anti-VEGF-receptor-2 antibody DC101 modulates NPs andnitric oxide delivery with nanocarriers	[105,117,118]
	Regulation of stroma	Extracellular matrix (ECM) targeting		[92,94]
		ECM synthesis	Metelimumab (transforming growth factor-β ligand-blocking antibody) conjugate NPs can enhance loaded drug effectivity. Prolyl-4-hydroxylase inhibitors (which inhibit collagen synthesis in vascular smooth muscle cells) conjugate NPs can enhance drug effectivity	[123,124,125,126,127,128]
		ECM degradation	Inhibition of hyaluronidases, collagenase enzymes, and putrescine regulates ECM degradation. Conjugating these particles into the loaded NPs can enhance the drug’s effectivity	[122,130,131]
		ECM signaling	Volociximab inhibits angiogenesis by interfering with integrin α binding with fibronectin in tumor vasculature. Co-administration of volociximab with other tumor-mimicking drugs can be a more effective therapeutic target	[123,129]
		ECM mimicking	Preparing artificial extracellular matrix (AECM) based on transformable laminin (LN)-mimic peptides and hydrogel-fabricated NP-loaded drugs can enhance effectivity	[92,132,133]
		Reducing cancer-associated fibroblast (CAF) activity		[92]
		CAFs disruption	Vimentin expression increased the migration and invasion of cancer cells. Preparing artemisinin (which inhibits vimentin expression) as a capping agent for the NPs loaded with drugs can be useful. Fibroblast activation protein is overexpressed in the stroma. *N*-(4-quinolinoyl)-Gly-(2-cyanopyrrolidine)-capping NPs inhibit FAPs overexpression with the active drug, which may be useful to regulate stroma	[134,135,136,137,138]
		Reprogramming CAFs	CAFs act as either immune suppressive or supportive agents. Angiotensin receptor blockers (ARB) reduce latent CAF activity. ARB nanoconjugates can enhance immune-supportive activity	[139,140]
	Hypoxia manipulation	Oxygen supply elevation	Using theranostic conversion nanoprobe MnO_2_ NPs. In the tumor cell, excessive amounts of H_2_O_2_ and lactic acid are produced. Theranostic MnO_2_ reacts with acidic H_2_O_2_ and produces Mn^2+^ and enhanced O_2_ production	[141,142]
		Decreases oxygen consumption	Encapsulating photothermal therapy with electron transport chain hindering agents through NPs	[92,140,145]
		Using hypoxia-activated prodrugs (HAP)	HAPs are activated through spontaneous electron oxidoreductases. HAP agents combined with targeted therapy with checkpoint blockers increase the influx into the hypoxic zone	[143,144,145]
	pH manipulation	Acidity neutralizing agents	Sodium potassium citrate increased blood HCO_3_- levels in oral doses and neutralized the TME pH	[146,147,148,149]
		Controlling pH regulatory enzymes	As in the tumor microenvironment, acidic pH affects the chemotherapeutic drug efficacy. By regulating pH, the efficacy can be enhanced. Few drugs are carbonic anhydrase IX/XII and proton pump inhibitors	[146,147]
	Immunity modulation	Tyro3, Axl, and Mertk receptor (TAM) regulation	TAM overexpression increases cell survival and decreases apoptotic signaling. Again, TAM down-streaming promotes metastasis via migration and invasion. The immunosuppressive nature of the TAM arises from the polarization of macrophages M1 to M2. M2 releases immune-suppressive cytokines. M2 blocking agents and M1 reprogramming agents can regulate this immunity suppression. Using small-molecule tyrosine inhibitors and TAM receptor targeted ligands is useful to regulate it	[152,153,154,155]
		Regulatory T-cell (Treg) inhibition	Treg cells regulate T-cell immune responses to maintain cell homeostasis. But in the TME, Treg cells decrease the entry of T cells. Transforming growth factor-β (TGF-β) inhibitors and anti-PD-L1 antibodies reduce the TGF-β signal to promote T-cell infiltration into the TME. Again, cytotoxic T-lymphocyte-associated protein-4 (CTLA-4) antibodies remove the Treg cells and can enhance T cell functions	[152,156]
		Myeloid-derived suppressor cell(MDSC) inhibition	MDSC induces immune suppression by inhibiting T-cell, NK-cell, and macrophage functions. Targeting and inhibiting phosphatidylinositol 3-kinase (PI3K)δ, PD-L1 or CTLA-4, and multi-kinase MDSC can be controlled	[157,158]
Enhancement of active targeting		Surface ligand modification	Folate discs enhanced the permeability and photothermal efficacy	[153,166]
		Biomimetically modified NPs	Cancer cell membrane-coated NPs can carry antigens and drugs to the target	[164,165]
Tumor microenvironment responsive drug delivery system	Enhanced tumor penetration of carrier NPs	Functional moieties sensitive to a variety of Tumor cellular stimuli	In response to the TME, supramolecular architectures based on peptides can convert structurally and allow therapeutics for controlled release. This dissertation emphatically introduces peptide assemblies with a stimulus-responsive structural conversion to acids, high temperatures, and high oxidative potentials in tumor tissues	[153,166,167]
		Particle size modification	After blood circulation, particles with a large size can shrink in size due to internal stimuli, such as enzymes, acidic pH, and hypoxia. Using peptides or other favorable ligands, NP entraps to form corona at TME	[170,174,175,176,177,178,179]
	Enhancement of cellular uptake	Conversion of surface charges	It helps to eliminate long circulation times and cellular uptake by modifying its surface charge. An example is a pH-sensitive PEG coating	[180]
		Detachments of shell of the NPs	As nano-vectors accumulate at tumor sites via the EPR effect, overexpressed MMP-9 can detach the PEG corona to expose peptide RGD to facilitate cellular internalization	[92]
	Elevate the drug release at cancer site	Polymer switches between hydrophilic–hydrophobic triggered by TME signals	Protonation and de-protonation polymers present in the NPs can switch from hydrophilic to hydrophobic and trigger drug release at the targeted sites. Poly(2-(diisopropylamino)ethyl methacrylate) can trigger the drug release	[181,182]
		Cleavage with a sensitive linker	Hypoxia-sensitive linker	[144,181]

## 5. Novel Nanocarriers Based Treatment Approach

Cancerous cell proliferation and migration profiles are different from those of normal cells. A therapeutic dosage form should enter the TME to control cancerous cell proliferation and migration. The penetration of conventional dosage forms into the TME is less due to its heterogeneity and the above-mentioned other factors. In addition, traditional drug delivery systems are less specific for cancer cells. Due to the lack of specificity and less penetration into the TME, the required concentration of the drug does not reach the cancer cells. Non-eliminated cancer cells alter metabolic signaling pathways and drug metabolism, inactivate drugs, suppress apoptosis, alter epigenetics, change drug targets, enhance DNA repair, alter epithelial–mesenchymal transition, and enhance gene amplification. As a result, cancer cells cause MDR, survive, rocket, and migrate [60,183,184,185,186,187].

As the nanocarriers have a diverse range (from 01–1000nanometers) and can be tuned according to the requirements of the (<200 nm) targeted site, the study and use of nanocarrier-based targeted drug delivery have increased. Again, the nanoparticulate nanocarriers can incorporate multiple targeting agents to enhance bioavailability, drug delivery, absorption, targeting precision, and stimulus technique. Understanding and identifying cancer cells’ physiochemical behavior can help optimize nanocarriers. In addition, the release pattern of drugs from nanocarriers determines the effectiveness of nanocarrier-based drug delivery systems [60,183,187]. 

Nanocarriers can be classified as organic, inorganic, or hybrid based on the components used in their development [188]. 

### 5.1. Organic Nanocarriers

The biocompatible nano-structurally dispersed, versatile, and less toxic organic carriers can be synthesized through either non-covalent or covalent interaction between the drug and adjuvants. Almost all organic nanocarriers contain carbon as a primary component. Commonly used adjuvants for organic nanocarrier synthesis are lipids, polymers, surfactants, proteins, and polysaccharides [189]. Depending upon the compound used for the physical synthesis of organic nanocarriers for delivering the drugs to the lung, they are classified into lipid-based nanocarriers (solid lipid NPs, liposomes, micelles, and lipid nano-capsules) and non-lipid-based nanocarriers (mesoporous NPs, polymeric NPs, dendrimers, and metallic NPs) [189,190].

#### 5.1.1. Lipid Based Nanocarriers

As a carrier system, lipid NPs are biocompatible and biodegradable, and their toxicity is lower than that of polymeric NPs. In addition, it improves solubility and absorption to enhance bioavailability and pharmacokinetic parameters. These lipidic nanocarriers are classified into solid lipid NPs (SLN), nanostructured lipid nanocarriers (NLC), liposomes, lipidic nanocapsules, and niosomes [191]. In a recent clinical update, ceranib-2, a ceramidase inhibitor-loaded lipid NP, increased penetration through the membrane and bioavailability [192], as shown in Table 2.

##### Solid Lipid Based NPs

**Figure 4:**
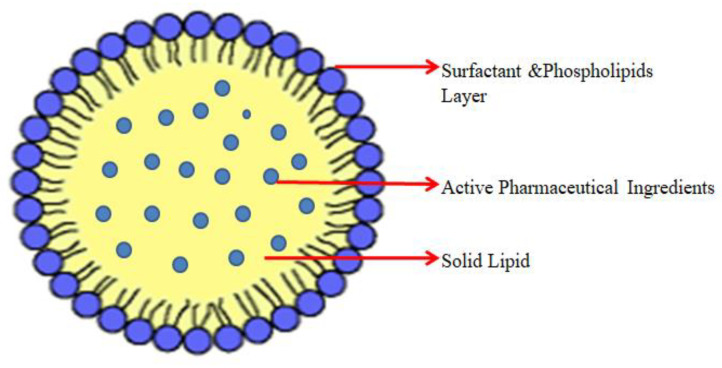
Solid lipid NP.

Solid lipid nanoparticles (SLN) are a surrogate of the colloidal drug delivery system, which can carry lipophilic and hydrophilic drugs, nucleic acids, and proteins to the targeted site. The size range of SLNs is 40–1000 nm [193]. It is a versatile, biocompatible, stable nanocarrier system with less toxicity. It is suitable for both active and passive targeting. Solid lipid NPs are prepared by dispersing the melted solid lipid in water, followed by the addition of emulsifying agents through different homogenization techniques or micro-emulsification. Supercritical fluid, solvent emulsification/evaporation, double emulsion, and spray drying methods can be used to prepare SLNs [190]. Primary solid lipids used in the SLN preparation are fatty acids, mono-, di-, triglycerides, or waxes. These biodegradable lipids of SLN can offer sustained release of drugs deep into the lungs and are for the pulmonary drug delivery system. Solid lipid NPs have a larger surface area and can load higher doses of active medicament. As per the requirement, in SLN, the drug can be incorporated into the matrix, shell, or core as shown in the Figure 4. SLN can be used in the preparation of oral dosage forms. Recently, studies have shown the higher transfection efficacy of cationic SLNs for the p53 gene targeting lung cancer [194]. A high-melting-point triglyceride in the SLN formulation is more efficient in the tumor cell environment [195]. Clinical updates indicate that folic acid-modified silymarin SLN enhances internalization through folate receptors in TME [196], as shown in Table 3. The main disadvantages of SLNs are their lower drug-loading efficacy and drug expulsion during storage. It can be rectified by mixing lipids with oil in a 70:30 to 99.9:0.01 ratio. SLNs can be optimized further by using appropriate ligands to overcome the TME, other than passive targeting [190,197,198].

In a study, researchers found that inhalable epirubicin-loaded SLN caused more cytotoxicity than epirubicin solution in the A549 cell line [199]. SLN loaded with docetaxel also prevented tumor growth and lung metastasis in 4T1 murine mammary carcinoma cells [200]. In a study, researchers found that the dual drug curcumin and paclitaxel-loaded SLN showed the highest tumor inhibitory action (78.42%) in the A549 cell line compared to other cell lines rather than the drugs separately administered. As well as enhancing P-glycoprotein efflux, this formulation reverses the MDR pathway and down-regulates NF-kB [201], as shown in Table 3. Enhanced green fluorescence protein plasmids and doxorubicin-loaded transferrin-conjugated SLN show improved anticancer activity [202], as shown in Table 4.

##### Liposomes

**Figure 5:**
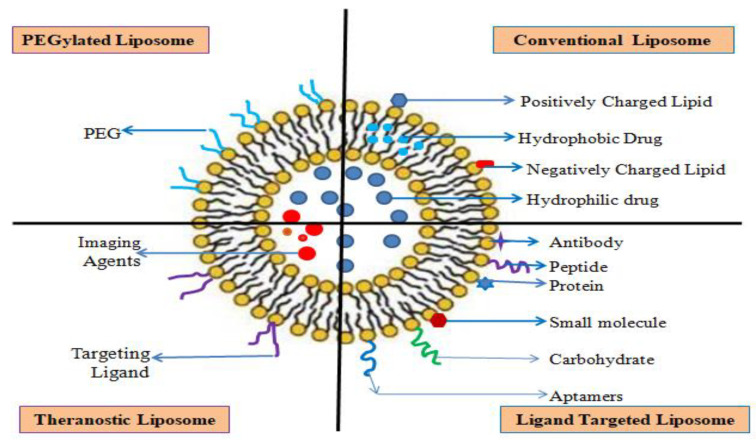
Liposome.

Liposomes are spherical vesicles with an aqueous core surrounded by natural phospholipids or synthetic amphiphiles and sterols in one or more bilayers with particle sizes ranging from 25 to 2500 nm, as shown in the Figure 5 [203]. This lipid-based drug delivery carrier is suitable for hydrophilic and lipophilic drugs as it has aqueous and lipidic layers. It can deliver macromolecules such asDNA, proteins, imaging, and active chemotherapeutic agents. It is a non-toxic, stable, high-vascular-density, and adjustable surface nanocarrier with a higher retention time in the targeted site. The half-life of this bilayer formulation is short in the systemic circulation. The preparation of liposomes generally begins with drying lipids from organic solvents and dispersing them in aqueous media, followed by purification and analysis. The composition of a bilayer determines the rigidity, fluidity, and charge of the layer. Long-chain acyl-functional phospholipids form the rigid, impermeable bilayer structure of the liposome. Unsaturated phosphatidylcholine shapes a flexible, permeable liposome. The commonly used phospholipids in liposome preparation are phosphatidylethanolamine and phosphatidylcholine. Microfluidizers, membrane extrusion, sonication, and homogenization techniques can control liposome size and size distribution. This nanocarrier NP can be used for active, passive, pH, magnetic, stimuli-responsive, and thermo-responsive targeting. Liposomes can enhance the loaded drug’s efficacy at the targeted site, therapeutic index, and stability. It also reduces the loaded drug’s toxicity and exposure to sensitive tissue [197,204,205]. Biofunctionalization liposomes enhance loaded drug efficacy in resisting lung cancer therapy through active targeting, as shown in Table 2 [206]. Again, in another clinical update (Table 2), researchers found that irinotecan and veliparib-loaded nano-liposomal intravenous formulations show combinational synergy for PARP and topoisomerase-1 inhibition along with better efficacy [207]. The disadvantages of liposomes are lower solubility, a shorter half-life, leakage of encapsulated drugs, oxidation and hydrolysis, and a higher production cost. Limitations and benefits of liposome drug carriers depend on liposome interaction with cells and their fate in vivo after administration. The interactions of liposomes with the cell surfaces take place either through adsorption or endocytosis. A liposome can be classified according to its functional modification: Conventional, PEGylated, ligand targeting, and theranostic [197,204,205,208,209]. These differently modulated liposomes can overcome the biophysiochemical difficulties of the active medicaments to reach the targeted sites. As well as liposome-loaded drugs suppressing the TME, soluble mediators in liposomal drug delivery systems inhibit TME immunity [208,209].

##### Conventional Liposome

Conventional liposomes and first-generation liposomes consist of an aqueous core encased in lipidic bilayers of cationic, anionic, or neutral phospholipids and cholesterol as shown in the Figure 5. Commonly used lipids and phospholipids for the preparation of conventional liposomes are 1,2-di-stearoryl-sn-glycero-3-phosphatidyl choline (DSPC), sphingomyelin, egg phosphatidylcholine, and monosialoganglioside. The main disadvantage of conventional liposomes is that they are rapidly eliminated. It occurs due to plasma opsonization and sequestration by reticuloendothelial macrophages [195,200,201]. In a study reported in Table 3, the researchers found that Honokiol-loaded liposomes show antitumor activity and induce apoptosis through the degradation of HSP90 client proteins to inhibit Akt and Erk1/2, which are mutant or wild-type EGFR signaling cascade effectors [210]. In another study reported in Table 3, researchers found that nano-liposomes loaded with the antioxidant Chinese herbal drug baicalin after intravenous administration to rabbits showed the highest drug accumulation in the lung and a higher survival time than the baicalin solution [211].

##### PEGylated Liposome

The sterically stabilized PEGylated liposomes were developed to improve stability and systemic circulation time by blending a hydrophilic polymer-polyethylene glycol (PEG), into the liposome preparation as shown in the Figure 5. Encapsulated PEG in the liposome formulation improved the efficacy of the entrapped drugs by creating a steric barrier that enhanced permeability and retention in the TME. PEGylating liposomes can overcome the opsonization of serum components, rapid recognition, and uptake by the reticuloendothelial system. It accumulates the active medicaments in the circulation and enhances the circulation time. If the accumulation increases exponentially, it interacts with other sites and causes toxicity [204,209]. It acts through passive targeting. According to a study, PEGylated liposomes accumulate in tumors via the EPR effect, but the mechanism of action differs in drug-resistant tumors. In hyper-permeable drug-resistant tumors, PEGylated liposomes penetrate deep into tumor cells. In hypo-permeable tumors, it enhances the proximity of tumor vasculature, thereby inhibiting angiogenesis [212,213]. The PEGylated NPs containing doxorubicin circulate longer with a higher intratumoral drug concentration, resulting in better therapeutic results [214]. A new study reported in Table 4 found that paclitaxel-loaded PEGylated liposomes enhance the efficacy of paclitaxel and reduce neuropathic pain associated with paclitaxel [215].

##### Ligand Targeted Liposome

The ligand-targeted liposome is a unique type of liposome in which monoclonal antibodies, proteins, growth factors, glycoproteins, and carbohydrates are chosen to couple with the liposome to target according to the overexpression at the disease site in an active targeting manner. Antibodies or fragments of antibodies are highly selective for this nanocarrier. In addition to entrapping more active drugs, it can act as a sustained-release agent at the target site. To improve the efficacy of the ligand-targeted liposome selection of targeted receptors, internalization versus non-internalization behavior of antigen, ligand selection, therapeutic agent selection, and location of the targeted site play an important role. This nanocarrier system can be classified as antibody fragment-targeted, receptor-targeted (folate receptor, transferrin receptor, sigma receptor), peptide-targeted, or multidrug resistance reversal-targeted as shown in the Figure 5. This nanocarrier is accessible if the target is in the blood and lymph nodes [216,217,218]. As shown in Table 4, the administration of the lipid stearic acid peptidomimetic conjugate SA-5 with doxorubicin ligand-liposome results in an antiproliferative effect in human epidermal growth factor receptor-2-mutated NSCLC [219]. Recently, researchers have tried dual ligand (CPP33 peptide and monoclonal anti-CA IX antibody)-modified liposome encapsulation with organic hetero-heptacyclic triptolide. They found that the dual ligand-modified liposome loaded with triptolide increased the cytotoxicity of triptolide with tumor-specific targeting and penetration without causing systemic toxicity, as shown in Table 4 [220].

##### Theranostic Liposome

Theranostic liposomes are hybrid liposome dosage forms that combine diagnosis profiles with targeted therapy to create a tailored treatment profile as shown in the Figure 5. Besides imaging purposes, it protects from systemic clearances. Commonly used nanosize imaging agents to prepare this type of liposome are iron oxide, quantum dots, and gold NPs. Here, imaging agents are covalently bonded to liposome surfaces. The active drugs are encapsulated at the core or embedded in the lipophilic bilayer shell [221]. In a study, researchers found that a dual-layered liposomal–gold liposome induces photothermal effects in cancer cells, as shown in Table 3 [222]. Another study revealed that folate-targeted theranostic liposomes containing paclitaxel and vinorelbine prevented NSCLC metastasis and cancer proliferation (Table 4) [223].

##### Micelles

**Figure 6:**
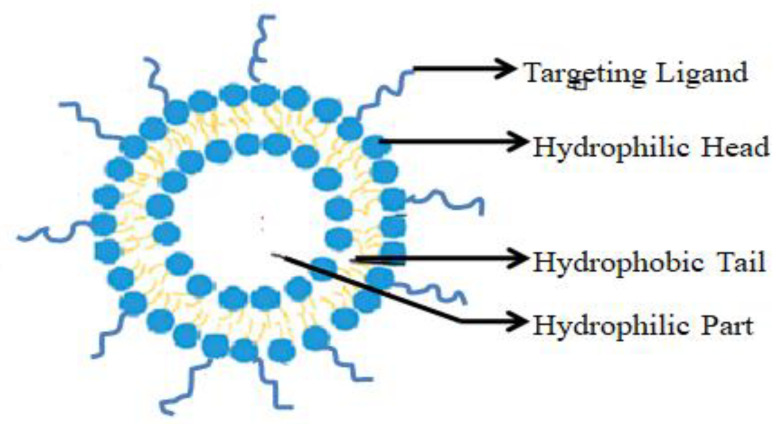
Micelle.

A micelle is a supramolecular assembly of surfactant phospholipids in water to form a colloidal dispersion. It has both hydrophilic and hydrophobic parts as shown in the Figure 6. The hydrophilic group exists in the center, whereas the hydrophobic group exists in the external solvent. In the inverse type of micelle, the hydrophobic group exists in the center, whereas the hydrophilic group exists in external solvents. In terms of structure and function, self-assembling micelle structures are similar to biological transport systems in that they can protect insoluble hydrophobic drugs. This type of nanocarrier can carry low-molecular-mass hydrophobic drugs, proteins, and genes. As the micelle has a size range of 50 nm, it helps deliver the drugs to the systemic circulation through tissue penetration and cellular uptake for active medicament accumulation, permeability, and retention. This can improve the encapsulated drug’s delivery to the targeted site. But the main disadvantage of the micelle drug delivery system is its shorter stability and premature drug release when it comes into contact with the systemic circulation as it dilutes. Using a covalent crosslinking strategy such as corona formation around the micelle, dimerization, and di-functional crosslinking can stabilize it. Another method to stabilize the micelle is the complexation of the micelle core [224,225]. One study has found that the micelle reprogrammed the CAFs to modulate the entry of APIs into the TME [226]. As reported in Table 2, pH-sensitive epirubicin conjugated micelles with anticancer drugs synergistically enhance the efficacy of epirubicin in resistant and metastasizing cancer [227]. In 2007, a few researchers reported that cremophor-free paclitaxel-loaded PLGA-b-methoxy PEG polymeric micelles with cis-platin showed better efficacy for the advanced stage of NSCLC [228]. Furthermore, researchers found that PLGA-PEG-maleimide micelles prepared by microfluidics exhibited a higher degree of cytotoxicity in NSCLC when loaded with docetaxel, as reported in Table 3 [229].

##### Lipidic Nanocapsule

**Figure 7:**
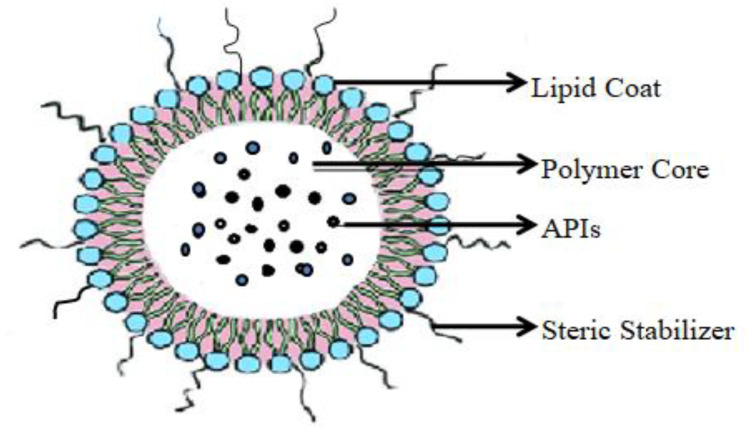
Lipid nanocapsule.

A lipidic nanocapsule is a hybrid biomimetic nanocarrier. It comprises medium-chain triglycerides encased in an aqueous phase using a PEGylated surfactant as shown in the Figure 7. Sometimes lecithin and co-surfactant are also used to prepare lipidic nanocapsules. Both active and passive targeting are possible with this form of the nanocarrier. Lipidic nanocapsules can incorporate both lipophilic and hydrophilic drugs. It can show adjuvant effects such asp-glycoprotein inhibition to favor higher anticancer APIs in the TME. Modification with a ligand can enhance the lipidic nanocapsules’ efficacy [230,231,232]. This formulation has better physical stability and a smaller particle size distribution (20–100 nm), which can be used for drug delivery through different routes. The disadvantages of lipid nanocapsules are the low encapsulation capacity of lipophilic drugs, leaky vessels, and instability in biological fluids [231,232]. According to researchers, lipidic nanocarriers containing erlotinib and modified with PEG polypeptide are cytotoxic to lung cancer cell lines HCC-827 and NCI-H358 (Table 4) [233]. Another study revealed that tretinoin-encapsulated lipid nanocapsules can overcome tretinoin resistance in the A549 cell line (Table 3) [234].

##### Nanostructured Lipid Nanocarrier

**Figure 8:**
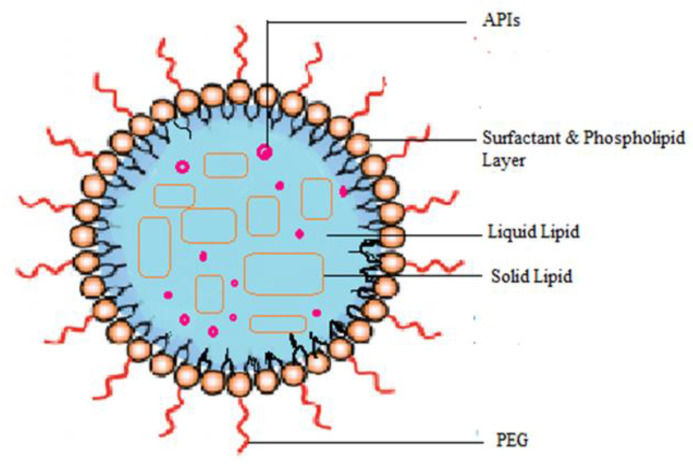
PEGylated nanostractured lipid nanocarrier.

Nanostructured lipid carriers are biocompatible, unstructured nanocarrier systems made from biocompatible lipids, surfactants, and co-surfactants as shown in the Figure 8. To prepare NLCs, solid and liquid lipids are mixed at 70:30 to 99.9:0.1 with surfactants ranging from 1.5% to 5% (*w*/*v*). Commonly used lipids for the formulation of NLCs are fatty acids, glycerids (mono, di, and tri), steroids, and waxes. To prepare NLCs, micro-emulsification, solvent displacement methods, and high-pressure homogenization techniques are used. The average size of NLCs is 40–1000 nm with a spherical morphology. The selection of lipids and surfactants can influence the physicochemical properties and quality of the materials, including particle size and drug loading. Due to its stability, high drug load, P-GP efflux inhibition, and diverse theranostic capabilities, it became a topic of interest for drug delivery studies [193,235,236,237]. A549 cells treated with transferrin ligand-conjugated NLCs entrapped in a plasmid containing enhanced green fluorescence protein demonstrated better efficacy for gene therapy in lung cancer treatment, as found by a few researchers (Table 4) [238]. In another study, an inhalable drug delivery system using NLCs loaded with doxorubicin, siRNA, and conjugated LHRH peptide showed better control of cancer cells than intravenous injections (Table 3) [239]. Further, researchers found that NLC fabricated with doxorubicin and sorafenib can stimulate PD-1 expression, down-regulate Treg cells, activate effector T cells, and regenerate the immune response (Table 4) while inhibiting esophagus tumors by bypassing the TME [240].

#### 5.1.2. Non-Lipid-Based Organic Nanocarriers

Non-lipid-based nanocarriers are the newer classes of drug delivery systems. The need for non-lipid-based drug delivery arises as the lipid nanocarrier has limitations such as hydrophilic drug loading, accumulation at non-targeted sites (spleen and liver), and reticuloendothelial clearance. Further, this new class has advantages such as multiple therapeutic targeting, higher toxicity, etc. Depending upon the particle used for the preparation, these non-lipid organic nanocarriers are classified into polymeric, dendrimers, mesoporous, and metallic NPs [197].

##### Polymeric Nanocarriers

**Figure 9:**
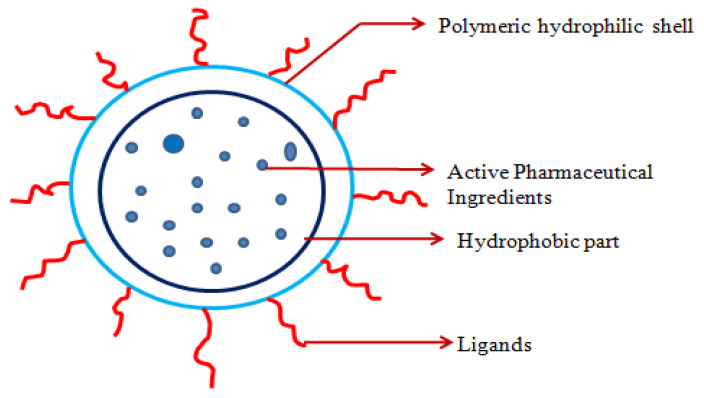
Polymeric NP.

Polymeric NP carriers are small (1–1000 nm), adjustable, rapidly absorbable, and versatile colloidal carrier systems to control the release of the entrapped active drug within the polymeric shell. Polymeric NPs can be classified into polymeric nanocapsules (reservoir systems) and nanospheres (matrix systems). Preparation methods for polymeric NPs include solvent evaporation and diffusion, nanoprecipitation, and reverse salting. Generally, the nanoprecipitation method is used to prepare polymeric nanocapsules. The stability of this nanocarrier depends on the adsorption of the active medicament onto the NP surface and the presence of surfactants as shown in the Figure 9. Microbial contamination is one of the challenges of this type of formulation. This problem can be resolved by adding preservatives, spray drying, or lyophilization. The drug delivery system is suitable for cancerous cell treatment using drug–nucleic acid combinations. These NPs can induce antitumor immunity in CD8+ T cells by regulating the lymphatic system and activating dendritic cells in TME [241,242,243]. The advantages of polymeric NPs include multiple therapeutic targeting and independent control of drug release. The main disadvantages of polymeric NPs are the synchronization of the pharmacokinetics and biodistribution of loaded compounds [241,242]. Novoselova M.V. et al. (2020) have found that the internalization of polymeric multilayer capsules in lung cancer cells is 75% higher than in healthy lungs. Embedding gemcitabine and clodronate in polymeric multilayer capsules inhibited macrophage-induced tumor growth (Table 3) [244]. In another study, silibinin, a low-water-soluble drug encapsulated in polycaprolactone/Pluronic F68 NPs, showed sustained release in the systemic circulation for up to 48 h, inhibited tumor growth, and improved the drug’s efficacy [245]. In a clinical update, the researchers found that polymeric NPs loaded with docetaxel can overcome drug resistance to refractory cancer (Table 2) [246]. Another clinical update, as shown in Table 2, is that polymeric micelles loaded with anticancer drugs are capable of releasing drugs whose AUC, Cmax, and volume of distribution are unstable [247]. As reported in Table 2, polymeric NPs entrapped with hypoxia-responsive photosensitizers and chemotherapeutic drugs produce reactive oxygen species that enhance the efficacy and photodynamic response of cancer treatments [248].

##### Dendrimers

**Figure 10:**
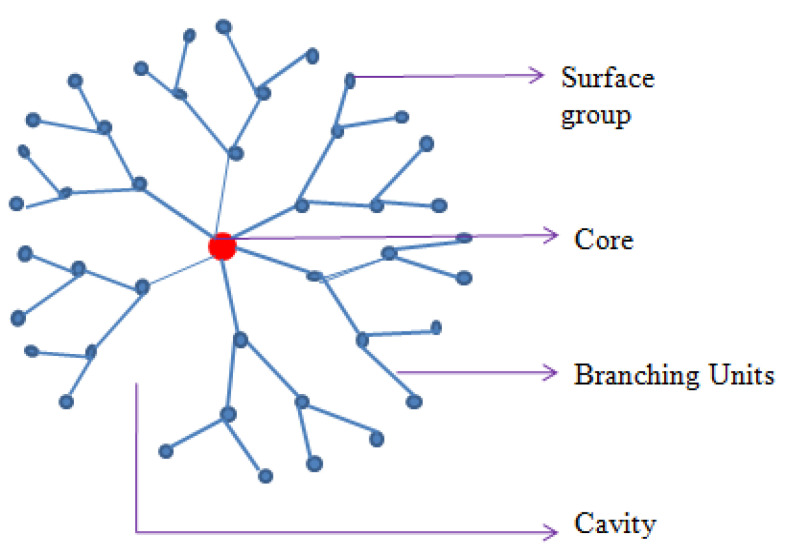
Dendrimer.

Dendrimers, arborols, or cascade molecules are a 1–100nm-size, three-dimensional, highly ordered, monodisperse, globular polymeric, symmetric macromolecular, hyperbranched macromolecular, tailored carrier system suitable for targeting drugs. The macromolecular dendrimers consist of a core, a repeating mantle, and a functionalized group corona [249,250,251]. Dendrimers’ tunable surfaces facilitate covalent modification to create stable micelle-type structures suitable for non-covalent encapsulation of APIs. Dendrimers are more stable under high shear stress, dilution, temperature, and pressure than liposomes and micelles [252,253]. It can act through both active and passive targeting. The PEGylation of dendrimers shields against attacking proteases and improves the water solubility of the dendrimers and their loaded drugs. The covalent conjugation of mAbs with the multivalent hydrophobic inner core (encapsulated with the APIs) of dendrimers can cause toxicity to the targeted cells as shown in the Figure 10. A few interesting biomedical dendrimers are polyamidoamine and polypropylene imine. The amine group of corona produces more toxicity and limits its use [249,250,251]. Poly(propylene amine), polyglycerol, and polyethylene imine dendrimers release APIs in a pH-dependent manner. Commonly, at pH 5–6, these dendrimers release the drug at a faster rate as compared to pH 7.4 [252,253]. Generally, dendrimers are prepared using either divergent or convergent methods. In both, the dendrimer grows outward from a multifunctional core molecule. The core molecule reacts with monomer molecules containing one reactive and two dormant groups. Then, the activated new periphery of the molecule reacts with more monomers. Encapsulation, electrostatic interaction, and covalent conjugation methods load drugs onto dendrimers [108,249,250,251].

Further, according to the need, a modified dendrimer can overcome the first-pass effect, immune clearance, cell penetration, and off-target interactions. These unique characters enhance the circulation time followed by the maximum amount of entrapped drugs to the targeted site [108]. Researchers found that siRNA and cis-di-amine platinum-loaded polyamidoamine dendrimers conjugated with folic acid nanocarriers for targeting H1299 performed better than individual therapies and showed negligible toxicity to normal MRC9 lung fibroblast cells (Table 4) [254]. Further, in a clinical update [Table 2], the researcher found that a dendrimer loaded with bromoenol lactone inhibitors improves the drug’s solubility, tolerability, and therapeutic index [255].

##### Polystyrene NP Carriers

**Figure 11:**
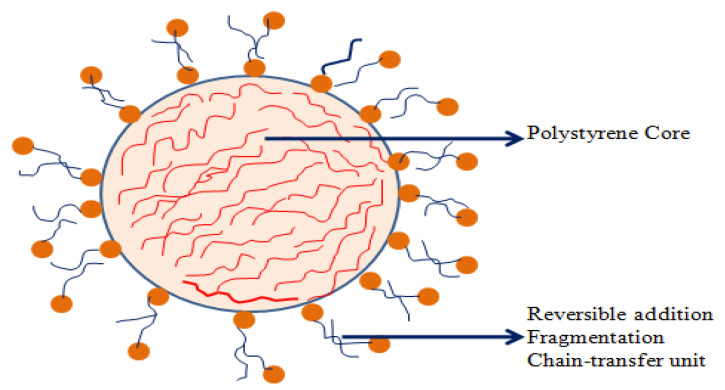
Polystyrene NP.

Polystyrenes are biocompatible polymers prepared by the polymerization of styrene monomers. As a result of external stimuli, it undergoes rapid and reversible phase transitions. It results in a desired drug release pattern from the formulation. Again, as per the previous reports, polystyrene had better penetration through the skin, respiratory tract, and digestive tract by forming a protein corona around it in the biological system. The Environmental Protection Agency report states that more than 1000 mg/m^3^ of chronic exposure to styrene is toxic for humans [256,257]. If the dose exceeds the limit, the chance of tumor formation increases, as styrene’s epoxide metabolite is genotoxic and can adduct DNA in humans [258]. Polystyrene NPs (10 nm) as shown in the Figure 11, can pass the blood–brain barrier and deposit in the alveoli, and their toxicity varies according to their size [259]. Polystyrene NPs can enhance reactive oxygen species production in the TME to inhibit cell growth [256,260]. It also disturbs metabolic activity, and amino-modified polystyrene causes cytotoxicity [261,262]. Further, no research suggests the carcinogenic nature of modified polystyrene NPs in a limited dose. Due to the complexity of drug delivery to the lungs, modified polystyrene NPs may be used. Research has found that polystyrene NP surfaces functionalized with carboxy, amino, and pristine accumulate inside cells and cause cytotoxicity and genotoxicity in A549 NSCLC [263].

##### Carbon Nanotubes

**Figure 12:**
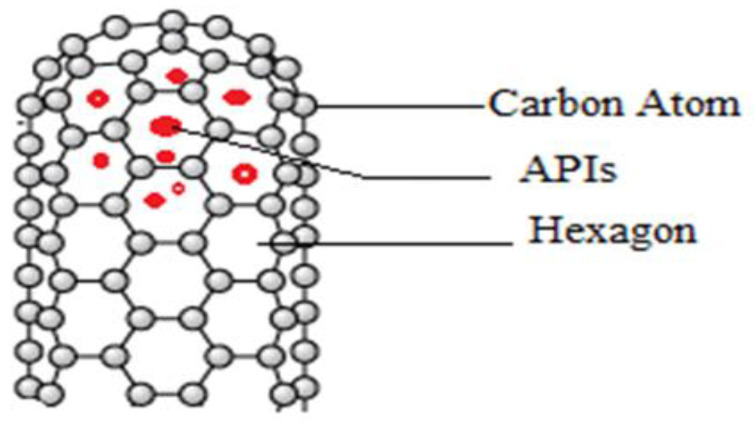
Carbon nanotube.

Carbon nanotubes, forged and rolledup with graphene, are cylindrical-shaped single-walled or multi-walled drug carrier NPs with an inner diameter of 0.4 to a few nm and an outer surface diameter varying from 2–30 nm as shown in the Figure 12. Low-temperature chemical vapor deposition (CVD) accurately synthesizes CNTs. After synthesis, purification of the CNTs is necessary to reduce larger graphite particles. CNTs have similar photothermal effects as other metal NPs. They have strong optical absorption due to their specific optical properties. To enhance drug loading, CNTs have π-electro-conjugated surfaces that interact with hydrophobic APIs. CNTs’ limitations as drug carriers include their lower aqueous solubility, physiochemical characteristic-dependent pharmacokinetic profile, and toxicity [82,264,265]. Generally, smaller-sized CNTs are not better suited for treating lung cancer, as they may cause pleural lesions, inflammation, pleural fibrosis, and malignant mesothelioma [266]. In addition to cancer diagnosis, CNTs can be usedin imaging, augmenting tissue engineering, and delivering drugs. Recently, efforts have been made to reduce toxicity by using CNT-based hydrogel [267]. Researchers reported that an ELISA test comparing untreated NSCLC A543 cells with multi-walled carbon nanotubes conjugated with bromocriptine was lethal (Table 4) [268]. In another study, researchers found that the higher concentration of multi-walled carbon nanotubes causes nuclear condensation and DNA laddering. It generates reactive oxygen species that induce oxidative stress and apoptosis in A549 cells [269]. In a clinical update of Table 2, researchers found that carbon nanotube-based anticancer drug delivery can suppress drug resistance [270]. Another clinical update found that a protein–CNT complex can bind to tumor vasculature endothelial cancer cells and destroy them through the PTT effect [271].

### 5.2. Inorganic Nanocarriers

Multidrug resistance (MDR) is one of the factors contributing to therapeutic failures in lung cancer treatment. A solution to the MDR lies in the use of inorganic nanocarriers. Nanocarriers can carry compounds with low molecular weights as well as imaging agents. Combining these nanocarriers with photothermal and interference agents can further enhance their efficacy. Their unique physiochemical properties, stability, inertness, and biocompatibility further improve their applications [272,273,274].

#### 5.2.1. Metallic NP Carrier

**Figure 13:**
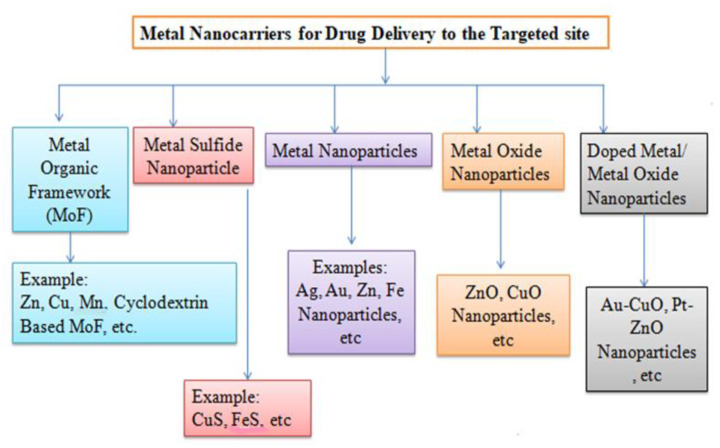
Metal NP.

Metallic nanocarriers are colloidal 10–1000 nm systems where the active medicaments are either dispersed or encapsulated in a shell or structure of a metal-based cavity or covalently attached to the surface of the metal cavity. These metal nanocarriers act as hypothermic agents. It reacts through surface plasmon resonance. Metallic NPs efficiently absorb near-infrared (NIR) light for photothermal therapy (PTT). So, it is an attractive option for cancer-targeted drug delivery. Again, surface modification of metal nanocarriers can optimize drug delivery to the targeted site. The metallic NP has diverse applications, from diagnosis to treatment. It can act via active and passive targeting in the cellular and subcellular regions. A drug’s efficacy at a targeted site depends on the physicochemical properties of metal nanocarriers. The main disadvantage of metallic NPs is their toxicity due to their accumulation in different tissues, which leads to stimulation or suppression of the immune response. It also causes acute toxicity through oxidative stress. The persistence of oxidative stress can cause DNA damage or genotoxicity. Different forms of metal NPs include pure metal NPs (gold, silver, copper, titanium, platinum, zinc, magnesium, iron, and alginate); metal oxide NPs (titanium dioxide, silver oxide, and zinc oxide); doped metal/metal oxide/metal nonmaterial; metal sulfide; and metal–organic frameworks (MOFs) nonmaterial, as shown in the Figure 13 [275,276]. A recent clinical study found that sorafenib-entrapped metal cluster-doped protein NPs enhance drug efficacy and bioavailability through enhanced optical contrast, magnetic contrast, and modulation of zeta potential [277]. Another study in Table 2 found that phospholipids containing cis-platin prodrug entrapped in MnO2 NPs generate a glutathione oxidation–reduction reaction to cause hyperpyrexia and activate photothermal effects to treat lung cancer [278]. In a recent clinical update, researchers reported that anticancer drug-loaded metal–organic frameworks cause dual effects of photothermal nature along with the inhibitory properties that can treat cancer [279]. Further, a clinical update of Table 2 found that antitumor drugs loaded in a hybrid metal–organic framework modified with cholesterol oxidase can catalyze the overexpressed cholesterol and overcome MDR [280].

##### Gold NP

**Figure 14:**
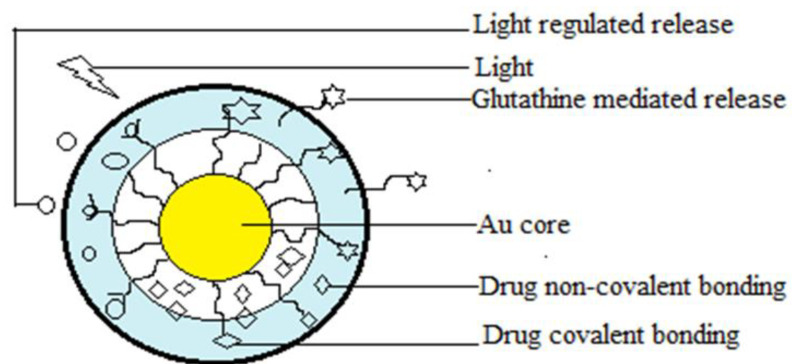
Gold NP.

Gold NPs (GNPs) are 5 to 400 nm in size and vary in shape as shown in the Figure 14; they are optoelectric, mildly antibacterial, and targeted drug delivery carriers. Their antibacterial activity depends on the intensification of ROS generation in the microbial cells. Other biomedical applications of GNPs are photodynamic immunotherapy for cancer treatment, diagnostic agents, etc. The photothermal activity of GNPs is due to the excitement of electrons when irradiated with laser light. GNPs can be synthesized using the bottom-up reduction method of chloroauric acid (HAuCl_4_). Commonly used reducing agents are sodium citrate, borohydride, polyalcohol, amines, etc. [281,282,283]. The reported absorption of GNPs in oral administration is low. IV administration of GNPs accumulates in the spleen, liver, and lung, and elimination is less. GNPs increase glucose and catalytic enzymes (alanine aminotransferase and aspartate transaminase). They also affect liver function [284]. In a study, researchers found that methotrexate conjugated GNPs in a lower dose inhibit tumor growth compared to methotrexate (without loading or conjugated) in Lewis lung carcinoma (Table 3) [285]. In another study, researchers reported significant cytotoxicity and apoptosis in lung cancer stem cells when aluminum (III) phthalocyanine chloride tetra sulfonic acid and anti-CD133 antibody bioconjugate GNPs were administered (Table 4) [286]. On the A549 cell line, researchers found that silibinin-conjugated gold NPs released pH-responsively enhanced silibinin efficacy up to 4–5 times (Table 3) [287]. In a recent clinical update of Table 2, researchers have found that T-cell, microRNA, or peptide-conjugated or entrapped gold NPs enhance the EPR effect and its photothermal nature to inhibit cancer cell growth [288].

##### Silver NP

Silver NPs are 1–100 nm-sized, stable, catalytic, and high-conductance NPs. It regenerates reactive oxygen species or releases silver ions from its surfaces. This NP has antibacterial, antifungal, and anti-inflammatory properties. NPs of silver penetrate the skin less than other inorganic metals [289,290]. Oral administration of silver NPs accumulates in different organs as silver ions, especially in the liver and spleen. The retention time of silver NPs in the brain and testis is longer [291]. In H1299 lung cancer cells, these NPs can cause cell apoptosis and inhibition of nuclear factor transcriptional activity in a dose-dependent manner [292]. Again, as reported, *Toxicodendron vernicifluum-modified * silver NPs can cause 82.5% of cancer cell apoptosis in A549 lung cancer cells [293]. In another study, as reported in Table 3, embelin biofunctionalized silver NPs exhibit significantly lower necrotic cells than apoptotic cells in A549 cancer cells in a dose-dependent manner [294]. In Table 3, as compared with cis-platin, *Juniperus Chinensis * leaf extract fabricated into biofunctionalized silver NPs showed better anti-proliferation and apoptotic effects on the A549 lung cancer cell line [295].

##### Platinum NP

Platinum NPs are variedly shaped (spherical, rods, tetrahedral, and cubes) and have a 2–100 nm stable brownish-red or black colloidal or suspension dispersion system. It acts as an automotive catalytic converter for hydrogenation in the chemical industry. In addition, platinum NPs are used as drug delivery and imaging agents [296,297,298,299]. In vitro, analysis of platinum NPs inhibits the growth of A549 cancer cells in a dose-dependent manner [300]. Again, blood-triggered platinum NP sactas an anticancer agent by forming the protein corona [301]. Another study revealed that platinum NPs reverse the oxidative stress in lung adenocarcinomas in the A549 lung cell line [299,300]. Again, biofabricated platinum NPs are biocompatible and have catalytic and anticancer activity [167,300]. In another study, researchers found that poly(lactic-*co*-glycolic acid) and polyethylene glycol-modified platinum NPs loaded with anti-EGFR showed better efficacy in triple-negative breast cancer [302]. Furthermore, porous Au–Pt NPs loaded with doxorubicin modified with c-RGD exhibit better drug release patterns and enhanced anticancer properties via photo-induced and photothermal processes. The scavenging activity of NPs enhances drug-induced oxidative stress (Table 4) [303].

#### 5.2.2. Metal Oxide NP

The redox-reactive metal oxides of a size range less than 200 nm are another choice of nanocarriers for antibiotic-resistant wound healing, along with growth factors. A metal oxide is stably tunable, has a high surface area, and is a porous NP with antimicrobial, antifungal, and antiviral properties [304,305]. Again, researchers have found that CuO, NiO, and Fe2O3 generate ROS in normoxia and hypoxia that cause toxicity to the tumor environment in lung cancer patients [306].

##### Zinc Oxide NP

Metal oxides such as ZnO are less than 100 nm rod, hexagonal, tripod, spherical, and different-shaped photographic catalytic metal oxide NPs that can absorb and reflect ultraviolet rays. This nature is helpful for bio-imaging purposes. NPs of zinc demonstrate biological activity such as apoptosis upon activation with light. The anticancer, antibacterial, and antimicrobial activity of zinc oxide NPs is due to excess ROS production. ZnO NPs cause cell apoptosis in colon carcinoma by altering mitochondrial IL8 release function [307,308,309,310,311]. The exact mechanism also works against NSCLC [154,310,311]. Zinc NPs loaded with cis-platin and gemcitabine enhanced the inhibition of tumor formation and the apoptotic nature of the drugs. The formulation also decreases the total cell viability in the A549 cell line, as reported in Table 3 [310]. In a recent clinical update in Table 2, researchers found that rapamycin-loaded zinc–organic frameworks work by inhibiting the mTOR pathway. It also enhances the sensitivity of chemotherapy [312].

##### Iron Oxide NP

Iron oxide NPs are highly reactive, rapidly oxidized (in the presence of oxygen and water), superparamagnetic, 1–100 nm NPs. Among its many uses, it is suitable for magnetic imaging, bimolecular separation, and targeted drug delivery. Three oxides of iron NPs are magnetite (Fe_3_O_4_), maghemite (γ-Fe_2_O_3_), and hematite (α-Fe_2_O_3_) [154]. Iron oxide NPs interact with the immune cells to modulate the immune response. Further, the PTT activity of iron oxide can act as an antitumor response [295]. Initially, iron NPs caused higher cytotoxicity due to the quicker release of Fe ions. A carbohydrate or polysaccharide (carboxymaltose) complexed with iron NPs can control the release of Fe ions to saturate transferrin. The action mechanism of this NP starts with the uptake by the RES system, followed by degradation of the polymeric or carbohydrate shell through macrophages to conserve it as ferritin or excrete it through ferroportin-1 [313]. Iron oxides can be used for cancer diagnosis purposes using their photothermal activity. With FDA-approved ferumoxytol co-incubated with macrophage treatment for metastatic lung cancer in the liver and lungs, early mammary cancers have shown caspase-3-mediated apoptosis. It also increases the pro-inflammatory response in M1 macrophages [313,314]. Researchers also found that the proliferation of lung cancer cells was slowed by SPIONs coated with silica monolayers, as shown in Table 3 [315]. In another study, researchers discovered that iron oxide nanoflowers loaded in thermo-sensitive fluorescent liposomes could enhance the efficacy of cytotoxic drugs against lung cancer by causing hypothermia [316].

##### Copper Oxide NP

Copper oxide NPs are 1-100-sized antimicrobial, antibacterial, catalytically reactive, high surface-to-volume ratio NP carrier systems prepared from copper salt in the presence of surfactant [275,317]. It can be prepared from plant extracts such as *Euphorbia nivulia’s latex*, *Magnolia kobus * leaf, *Calotropis procera * latex, etc. [318]. Copper oxide (CuO), the oxide NP of copper, is effective for cancer cells. Copper displayed a dose-dependent degradation of DNA molecules by generating oxygen [319]. Researchers have reported that copper oxide promotes anticancer activity in A549 cell lines via I, II, and IV HDAC mRNA expression [320]. Further, researchers have found that biofilm-producing bacteria and cancer cells do not tolerate actinomycetes mediating CuO NPs [321].

##### Titanium Dioxide NP

Titanium dioxide NPs are 1–100 nm-sized photocatalytic nanocarriers. Photocatalysis and self-cleaning mechanisms apply to optics, materials science, electronics, catalysts, pigments, and biology [322,323]. TiO_2_ NPs exhibit antimicrobial activity via photocatalytic free oxide and peroxide formation [322,324]. According to the researchers metal-doped TiO_2_ improves antimicrobial properties. It modifies light absorption to enhance photocatalytic properties [325]. The anticancer activity of TiO_2_ NPs is also due to the production of radical oxides to reduce oxidative stress. In a study, researchers reported that TiO_2_nanosquares, nanotubes, and fine particles have immunomodulatory effects. These TiO_2_ NPs inhibit tumor angiogenesis via proinflammatory responses [325]. According to Behnam MA et al.(2018), PEGylated TiO_2_ NPs destroy solid tumors through photothermal effects [326]. According to another study, TiO_2_ inhibits lung cancer proliferation, DNA damage, and apoptosis through the intrinsic mitochondrial pathway [327]. YSA peptide-conjugated mesoporous titanium peroxide NPs loaded with cantharidin produced reactive oxygen species and increased photodynamic lung cancer apoptosis [328]. In a recent clinical update reported in Table 2, researchers have found that antitumor drugs entrapped in bionic titanium dioxide synergistically generate reactive oxygen species to enhance the loaded drug’s efficacy [329].

##### Magnesium Oxide NP

A magnesium NP is typically a black, spherical NP with a size range of less than 100 nm and a specific surface area of 30 to 70 m^2^/g [330]. Among all magnesium NPs, magnesium oxide NPs (MgONP) have antibacterial activity in a concentration-dependent manner. It causes physical injury to the cell wall along with ROS production to damage the DNA. Magnesium oxide NPs also reduce the tobacco bacterial wilt index [331,332,333]. In addition to being antimicrobial and photocatalytic NPs, green MgONPs exhibit a high level of cytotoxicity against the MCF-7 breast cancer cell line [334]. MgONPs also form complexes with human serum albumin and induce cytotoxicity against K562 cell lines [335]. Using pH-sensitive polymer-coated Mg nanoflowers for photoacoustic and bubble-enhanced ultrasound imaging can break the polymer shell for hydrogen generation in acidic TME [336].

#### 5.2.3. Metal Sulfide NP

Photovoltaic, electrical, and optoelectrical metal sulfide NPs are varying size (01–100 nm) and shape (spheres, hexagons, cubic, etc.). Colloidal solvothermal synthesis can produce them [337,338,339,340]. These biocompatible NPs convert light, enhance radiation, and activate the immune system. Metal sulfides also enhance the Fenton catalysis process. It also enhances the EPR effect [341,342,343,344,345,346,347]. Recently, metal sulfides have been tried in cancer treatments using PTT, immunotherapy, radiotherapy, and targeted drug delivery mechanisms with limited success [339,344,345]. Recently, researchers found that PEG-surfaced copper sulfide NPs enhance immunotherapy efficacy via PTT and antigen capture [346]. Metal sulfide can be used for diagnostic purposes such as photoacoustic and multimodal imaging.

#### 5.2.4. Metal–Organic NPs

Metal–organic NPs are high-internal-surface-area-coordinated porous polymeric clusters of inorganic compounds with organic ligands. These NPs are suitable for chemical sensing, separation, drug delivery, catalysis, and storage. The surface modification of metal–organic frameworks using biofunctionalization according to their targeted sites can enhance their efficacy at the target sites. The prime challenge for synthesizing these NPs is the non-uniformity of the prepared NPs and the slow nucleation rate. A commonly used process to obtain uniformly smaller NPs in the range of 10–100 nm burst nucleation is adopted, followed by termination of precursors using depletion [347,348,349]. A newer treatment regimen for cancer is photodynamic therapy (PDT). Its effectiveness at the targeted sites depends on the presence of light, a photosensitizer (PS), and oxygen molecules. The porphyrinic metal–organic framework can naturally harvest light, transport oxygen, catalyze reactions, and transfer electrons to instrument PDT cancer therapy. PS-based metal–organic framework NPs with precise spatial arrangements can improve PDT efficacy. Bioconjugations with ligands to the metal–organic framework can enhance the selectivity of the NPs to the targeted site. Metal–organic frameworks can deliver chemotherapeutics and nucleic acids [347,348,349]. Recently, researchers found that RGD peptide biofunctionalized metal–organic framework loaded with doxorubicin enhances the loaded drug efficacy 4–5 times (Table 4) [85].

#### 5.2.5. Quantum Dots

**Figure 15:**
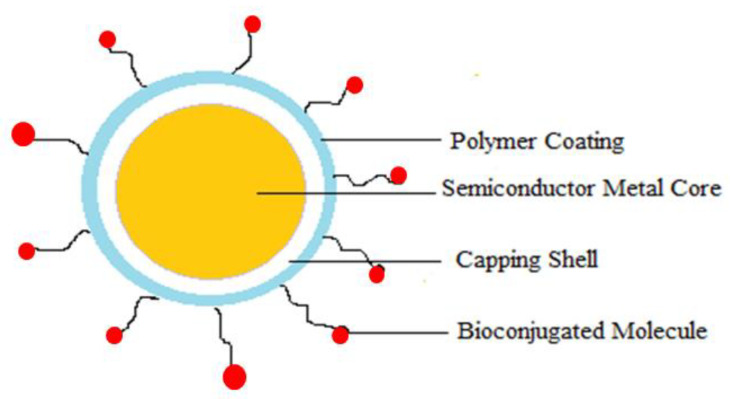
Quantum dots.

Quantum dots are colloidal electronic and fluorescent nanocrystals with an average diameter of 2–100 nm and unique electronic, physical, and photo-physical properties as shown in the Figure 15. It is known as an artificial atom due to its disjunctive electronic energy level. Unlike isolated atoms, artificial atoms have disjunctive electronic energy levels. As a semiconductor heterostructure, a quantum dot traps charge carriers (electrons and holes) in a volume approximately equal to the quantum mechanical wavelength of its components. Due to these properties of quantum dots, interest has increased in biomedical, bio-sensing, intracellular protein tracking, tissue engineering, drug delivery, and bioterrorism purposes. Again, in cancer treatment, drug delivery to the tumor microenvironment is challenging. Smaller quantum dots increase permeability, and a higher surface area enhances targeting efficacy [350,351]. Recently, researchers found that modified graphene QDs have higher accumulation in the hypoxia-induced oral squamous tumor cell microenvironment with low systemic toxicity [352]. Conjugating QDs with an active drug improved internalization in tumors resistant to drugs [353]. The QD nanocrystals modified with folic acid and 11-mercaptoundecanoic acid show cytotoxicity, genotoxicity, and migration-inhibitory activity against A549 lung cancer cells [354]. Hyaluronic acid-conjugated ZnO-based pH-responsive doxorubicin QD shows a synergistic effect on tumor growth, as shown in Table 4 [355].

#### 5.2.6. Magnetic NP

**Figure 16:**
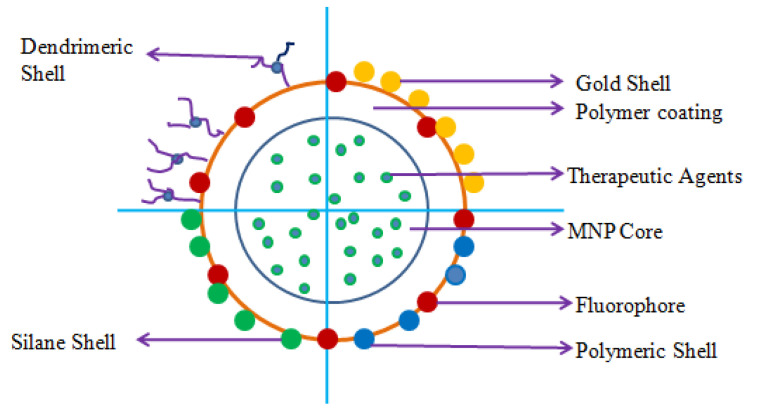
Magnetic NP.

Magnetic NPs (1–100 nm) are magnetic-field-manipulating NPs that connect to form clusters with magnetic nano-chains composed of magnetic materials (iron, nickel, and cobalt) and chemical functional components, as shown in the Figure 16. A magnetic NP consists of a magnetic core, a protective coating, and a surface functionality linker. Commonly used surface functionality linkers are synthetic organic polymers, silica, gold, and organic polymers [356]. Maghemite Fe_2_O_3_ or magnetite Fe_3_O_4_ magnetic NPs exhibit excellent MRI contrast properties. They also provide the required systemic toxicity and can be used as a catalytic nonmaterial, magnetic colloidal photonic crystals, resonance imaging, targeting biomedicine, etc. [357,358]. In cancer theranostics, the use of magnetic NPs has increased due to their larger surface area, smaller size, magnetic resonance imaging capability, ease of synthesis, ease of decoration, lesser toxicity, and better delivery vehicles. Different preparation methods may vary the shapes and sizes of magnetic NPs. A magnetic field can target and release entrapped drugs within a magnetic NP by altering its surface charges. As reported in Table 3, on an A549 lung cancer cell line, superparamagnetic iron oxide polymer (SPION) conjugated NPs loaded with doxorubicin are biocompatible without causing systemic toxicity [358]. Again, silica-coated SPION delays the proliferation of cancer cells [359].

#### 5.2.7. Ceramic NP

Ceramic NPs (CNs) are 50 nm diameter, temperature-resistive, inorganic nanocarrier systems prepared from metal (iron, calcium, titanium, silicon, etc.) oxides, carbides, phosphate, carbonates, albumin, and silica by successive heating and cooling. The preparation methods include sol-gels, low-temperature combustion, aerogels, hypothermal, Pechini-citrate gels, and microemulsions [360]. CNs can carry drugs that are enzyme, pH, or temperature sensitive. Besides photocatalysis, they can be used for dye degradation, imaging, and photodegradation. These CNs can be amorphous, hollow, porous, or polycrystalline [361]. Ceramic NPs can accumulate in the smaller capillaries, especially in the lung, and can cause risks in the circulation process. It can also affect the opsonization process. It can produce more reactive oxygen species and worsen the cancer environment. Mesoporous silica, calcium phosphate, carbon allotropes, and iron oxide are some of the ceramic NP carriers [362].

#### 5.2.8. Mesoporous Silica Nanocarrier

Mesoporous silica NPs are solid, tunable, and porous nanocarriers with high encapsulation capacity through endocytosis. These NPs have uniform pore size ranges of 2–6 nm. There are three types of MSNs—ordered MSNs, hollow MSNs, and core/shell MSNs. A hollow MSN can load more drugs than the others. Surface functionalization can enhance NPs’ physicochemical properties. A few techniques for preparing MSNs are growth quench, confinement techniques, separation of confinement, and growth techniques. Functionalization can be done with co-condensation, multifunctionalization, and grafting methods. The surface modification allows this NP to target both actively and passively [363,364,365,366]. Human cells are more likely to internalize 50 nm MSNs, although smaller particles exhibit longer circulation times. As particle size influences cytotoxicity, micrometric particles of 1 mm are less toxic than nanometric particles of 200 nm. Again, cationic NPs are more immunogenic and cytotoxic than neutral or anionic ones. In melanoma treatment, the FDA has approved multimodal silica NPs [363]. Conjugating ligands such as folic acid, DNA aptamers, transferrin, and antibodies with MSNs can enhance the efficacy of photodynamic targeted therapy for cancer. Researchers have found that MSN injection before anti-PD-1 resensitizes to overcome tumor resistance improves anti-PD-1 activity and protects immunity [366,367]. Researchers also found that siRNA co-delivered with chemotherapeutic drugs loaded in MSNs synergistically enhanced their efficacy and survivin protein inhibition [367,368]. In another study, folic acid-modified MSNs loaded with multidrug-resistant protein-1 siRNA and myricetin reduced cell viability, suppressed tumors, and up-regulated the expression levels of cleaved caspase-3 and PARP in the cancer cell lines A549 and NCI-H1299 [368]. In a clinical update of Table 2, researchers have found that an antitumor drug loaded in pH-responsive mesoporous silica-coated gold NPs can cause a photothermal effect in addition to the loaded drug mechanism to produce anticancer activity specifically in the tumor cells [369].

### 5.3. Hybrid Nanocarrier

The advantages and disadvantages of a variety of drug nanocarriers are discussed above. Recently, adding a combinational approach can mimic the disadvantages of nanocarriers and increase their efficacy. So, the concept of hybrid nanocarriers has arrived. These hybrid systems combine the benefits of different structural components to synergize the outcome of the therapy. Erosion and degradation are the processes by which the hybrid NP releases the entrapped active medicaments from the core. Multiple layers of lipids, polymers, and organic–inorganic compounds may protect the core materials, along with the solubility and permeability modifications of the entrapped active ingredients [370]. Recently, curcumin and survivin shRNA loaded in polymeric hybrid NPs with PLGA-conjugated triblock polymers (W5R4K-PEG2K-PHIS) showed better penetration into the TME and synergistic tumor suppression action [371].

**Table 2:** Patents of nanocarriers containing payloads and their clinical status in cancer.

Drugs	Nanocarriers	Dosage Form	Key Target	Approve Status	Approved By	Remarks	Patent No	Refs.
Ceranib-2	Lipid NP	Nanoemulsion	Ceramidase inhibitors	Approved	World Patent	Enhances penetration through the cell membrane and increases bioavailability	WO2020018049A2	[192]
Silymarin	Solid lipid NP	Intravenous injection	Folic acid	Pending	Chinese patent	Folic acid modified silymarin SLN enhances internalization in TME	CN111195239A	[196]
Anticancer drug	Liposome	Subcutaneous	Active targeting	Approved	Chinese patent	Biofunctionalization further enhances the loaded drug efficacy	CN105726483B	[206]
Irinotecan, veliparib	Nanoliposome	Intravenous	PARP and topoisomerase-1 inhibition	Granted	Japanese patent	Nano-liposomal formulation shows combinational synergy along with better efficacy	JP2018528184A	[207]
Anticancer drug	Epirubicin conjugated polymeric micelle	Intravenous and oral	Epirubicin resistant cancer	Granted	United States patent	pH-sensitive epirubicin-conjugated micelle with anticancer drug synergistically enhances the efficacy of epirubicin in resistant and metastasizing cancer	US10220026B2	[227]
Docetaxel	Polymeric NP	Intravenous	Drug resistant cancer	Granted	World patent	Refractory cancer	WO2014210485A1	[246]
Bromoenol lactone inhibitor	Dendrimers	Intravenous infusion	Inhibit bromoenol lactone	Granted	World patent	Bromoenol lactone inhibitor covalently attached dendrimers enhance the solubility, improve tolerability, and increase therapeutic index	WO2018154004A9	[255]
Anticancer Drug	Polymeric micelle	Intravenous	Endogenous protein	Granted	World patent	Facilitates drug release, especially in unstable, low AUC, low C_max_, high volume of distribution, critical micelle concentration above theoretical C_max_ of the drug	WO2014165829A2	[247]
Anticancer Drug	Carbon nanotubes	Parenteral administration	Drug resistance decreases	Granted	United States patent	Decreases drug resistance	US20150196650A1	[270]
Protein	Single-walled carbon nanotubes	Parenteral administration	Immune stimulant	Granted	United States patent	Bind to tumor vasculature and endothelial cancer cells	US20100184669A1	[271]
T cell	Gold NP	Systemic administration	T-cell receptor protein	Abandoned	United States patent	Conjugation or entrapment of the gold NP enhances the EPR effect, and then the photothermal effect inhibits the growth of cancer cells	US20140086828A1	[289]
Sorafenib	Metal-cluster-doped protein NP	Intravenous	EGFR	Granted	Worldwide	Metal cluster-doped protein NP enhances the drug efficacy and bioavailability by enhancing optical contrast, and magnetic contrast, modulation of zeta potential	WO2014087413A1	[277]
Phospholipids containing cis-platin prodrug	MnO_2_ NP	Intravenous	Multidrug resistant cancer	Pending	Chinese patent	Tumor cells carry platinum through endocytosis. In the uptake of drugs, MnO_2_ can generate a glutathione oxidation–reduction reaction to cause hyperpyrexia and activate photothermal effects to treat lung cancer	CN111214488A	[278]
Anticancer drug	Metal–organic framework	Intravenous	Double effects: Metal–organic framework photothermal effect Anticancer drug inhibits cancer through a specific mechanism	Granted	Chinese patent	Photothermal effects, in addition to the loaded drugs inhibitory action, can treat cancer	CN110652497A	[279]
Antitumor drug	Hybrid metal–organic framework modified with cholesterol oxidase	Intravenous	Catalyze the oxidation reaction of cholesterol	Granted	Chinese patent	Hybrid metal–organic framework can catalyze the overexpression of cholesterol and overcome multidrug resistance	CN112274648A	[280]
Photosensitizer and chemotherapeutic drug	Polymeric NP	Intravenous	Hypoxia responsive	Granted	Chinese patent	Hypoxic response polymer NP helps generate reactive oxygen species that enhance the chemotherapeutic drug’s efficacy along with the photodynamic response	CN108653288B	[247]
Antitumor drug	Mesoporous silica-coated gold NP	Intravenous	pH-responsive antitumor drug carrier	Granted	Chinese patent	Photothermal effect	CN107412195B	[370]
Rapamycin	Zinc–organic framework	Intravenous	mTOR pathway	Pending	Chinese patent	Inhibits mTOR pathway and enhances the sensitivity of chemotherapy	CN110693883A	[312]
Antitumor drug	Bionic titanium dioxide	Intravenous	Generate reactive oxygen species	Granted Drugive oxygen species	Chinese patent	Reactive oxygen species can enhance the antitumor drug’s efficacy	CN109646675B	[329]

**Table 3:** Non-functional nanocarriers in lung cancer.

Drug	Nanocarrier	Composition	Cell Line	InVitro Character Results			Remarks	Refs.
				Particle Size (nm)	Zeta Potential (mV)	Drug Release		
Paclitaxel + curcumin	Solid lipid NP	Hydrogenated soybean phospholipids; 1,2-distearoyl-sn-glycero-3phosphoethanolamine-N[methoxy(polyethylene glycol)-2000]; polyvinyl pyrrolidone k15	A549	121.8 ± 1.69	30.4 ± 1.25		Improved tumor inhibition. Reduces P-glycoprotein efflux, reverses MDR, and down-regulates the NF-κB pathway	[201]
Honokiol	Liposome	Sodium per-carbonate, cholesterol, PEG2000-DSPE	H1975, HCC827	130 ± 20	−20.0 to −30.0	Sustained manner	Shows time-dependent inhibition of degradation of HSP90 client proteins to inhibit Akt and Erk1/2, which are mutant or wild-type EGFR signaling cascade effectors	[209]
Baicalin	Nanoliposome	Phospholipon90H, Tween-80, citric acid, NaHCO3	A549	131.7 ± 11.7		Sustained release for 24 h up to 89.6 + 2.1%, stable for 12 months	Baicalin, the antioxidant, has antitumor activity	[210]
Gold	Theranostic liposome	Distearoyl phosphatidylcholine, 1,2-distearoyl-sn-glycero-3-phosphoethanolamine (methoxy(polyethylene glycol)-5000), 1,2-distearoyl-sn-glycero-3-phosphoethanolamine-*N*-[amino(polyethylene glycol)-2000], cholesterol		72.84 ± 22.49	−20 to −40	Sustained release	Liposomal gold liposomes act via photothermal effect, and their stability is also enhanced	[221]
Docetaxel	Micelle	PLGA-PEG-Mal	A549	72 + 1	Neutral	Sustained release	Higher cytotoxicity in NSCLC	[229]
Tretinoin	Lipidic nanocapsule	Poly(e-caprolactone), sorbitan monostearate, f polysorbate 80	A549	250	12.7 ± 0.9	Sustained release	Higher cytotoxicity through cell cycle arrest at the G1phase	[233]
Gemcitabine and clodronate	Polymeric multilayer nanocapsules	Poly-L-arginine hydrochloride, dextran sulfate sodium salt, tetramethylrhodamine isothiocyanate mixed isomers, rhodamine B, boric acid, glycerol, ethylene diamine tetraacetic acid disodium salt (EDTA), clodronate disodium tetrahydrate	A549	~250–500	Neutral	Sustained release	PMC inhibited macrophage-induced tumor growth	[244]
siRNA and different chemotherapeutic agents	Mesoporous silica NP		A549	172	-21	Sustained release	Combination of siRNA with chemotherapeutic agents shows synergistic effect with restraint of survivin effect	[368]
Silibinin	Polymeric NP	Silibinin (SB), polyvinyl alcohol (Mw 30,000–70,000 kDa), polycaprolactone (PCL), inhalable grade lactose	A549	108 ± 3.21–397 ± 3.19	Neutral	Sustained release	PCL/Pluronic F68 NPs loade silibinin significantly inhibited tumor growth in lung cancer-induced rats after inhalable administration	[243]
Methotrexate	Gold NP	Methotrexate, HAuCl4, sodium citrate, phosphate buffer 7.4	A549, QU-DB	14.3	−7.3 ± 2.5		Gold NP, through PTT effect, enhances the drug’s efficacy	[285]
Silibinin	Gold NP	HAuCl4, trisodium citrate dehydrate, silibinin, DMSO	A549	163 ± 5	−22.2 ± 0.458		Silibinin-conjugated gold NPs released pH-responsively enhanced silibinin efficacy up to 4-5 times	[287]
Embelin	Silver NP	Embelin, silver nitrate	A549	25	−5.42		Embelin-biofunctionalized silver NPs exhibit significantly lower necrotic cells than apoptotic cells in A549 cancer cells in a dose-dependent manner	[294]
*Juniperus chinensis* leaf extracts	Silver NP	*Juniperus chinensis* leaf extracts, silver nitrite	A549, HEK293	98.21 ± 1.54	−26.5		*Juniperus Chinensis* leaf extract fabricated biofunctionalized silver NPs showed better antiproliferation and apoptotic effects	[295]
Cis-platin, gemcitabine	Zinc NP	Zinc oxide NP, methanol, tri-ethylamine, cis-platin, gemcitabine	A549	21 ± 0.4	NA	Sustained release	NP loaded with cis-platin, gemcitabine inhibits tumor formation and enhances the apoptotic nature of the drugs	[310]
Iron NP	Iron NP modified with silica layer NP	Superparamagnetic iron (II,III) oxide NPs (SPIONs), tetraethyl orthosilicate, hexadecyltrimethylammonium bromide	A549BEAS-2B	101.3 ± 2.8	−26.1 ± 0.1	Sustained release	Delays the proliferation of cancer cells	[315]

**Table 4:** Functional nanocarriers in lung cancer.

Drug	Nanocarriers	Receptors	Ligand	Composition	Cell Line	In Vitro Character Result			Remarks	Refs.
						Particle Size (nm)	Zeta Potential (MV)	Drug Release		
Enhanced green fluorescence protein plasmid (pEGFP)+ doxorubicin	Transferrin-conjugated SLN	Transferrin	Transferrin	Enhanced green fluorescence protein plasmid (pEGFP)-N1 Soya lecithin Human transferrin	A549	267	42	Sustained	Improves anticancer activity	[202]
Paclitaxel	PEGylated large liposome	Blocks cell cycle in the G2/M phase	PEG	Lipo-Cat-PEG phosphatidylcholine, cholesterol, stearylamine, and DSPE-PEG2000	A549, LL2	180		Sustained	Antitumor activity with painful neuropathy reduction	[199]
Doxorubicin	Peptidomimetic conjugate (SA-5) liposome	Blocks human epidermal growth factor receptor-2 (HER2)	lipid stearic acid peptidomimetic conjugate SA-5	Lipid dipalmitoylphosphatidylcholine Poly(ethylene glycol) distearoylphosphatidylethanolamine Cholesterol	BT474 A 549 CALC3	107.19	−13.38 mV	Sustained	Antiproliferativeactivity	[215]
Triptolide	CPP33 peptide and monoclonal anti-CA IX antibody)-modified liposome	3D tumor spheroids	CPP33 peptide, monoclonal anti-CA IX antibody	Anti-CA IX antibody, CPP33 peptide with a terminal cysteine, soybean lecithin, NBD-DPPE, DSPE-PEG-MAL	A549	137.6 ± 0.8		Sustained	Tumor-specific targeting and increasing tumor cell penetrationwithout causing systemic toxicity	[220]
Erlotinib	PEGylated lipidic nanocapsule	EGFR	PEGylated polypeptide	Poly(ethylene glycol)-b-poly(L-aspartic acid), lecithin, sunflower oil, castor oil, Tween-20, and Span 20	HCC-827 and NCI-H358-20	∼200	−20	Sustained release	Higher cytotoxicity than erlotinib without loading in any nanocarrier	[233]
Plasmid-containing enhanced green fluorescence protein	Transferrin-nanostructured lipid carriers	Gene delivery	Transferrin	Soya lecithin, Maleimide-PEG2000-COOH, human transferrin (iron-free), stearic acid, L-a-phosphatidylethanolamine	A549	157	+15.9 ± 1.9	Sustained release	Gene targeting drug delivery	[238]
Doxorubicin, sorafenib	Folic acid Nanostructured lipid carrier	Immunotherapy	Folic acid	Folic acid, soya lecithin, maleimide-PEG2000-COOH, stearic acid		100		Sustained release	Helps overcome the TME, immune response enhancement, cytotoxicity	[240]
siRNA, *cis*-diamine platinum	Folic acid -conjugated polyamidoamine dendrimers	Folate receptor-α inhibition	Folic acid	Folic acid,	H1299, A 543	280	+14.5–17.2	Sustained release	Suitable for co-deliveryof si-RNA along with cytotoxicity	[254]
siRNA, myricetin	Folic acid conjugated mesoporous silica NP	Multidrug resistance protein-1, folate receptor	Folic acid	Folic acid, tetraethylorthosilicate, cetyltrimethylammonium bromide, myricetin	A549, NCI-H1299	109.9	Neutral	Sustained release	It accumulates in TME and prevents colony formation by enhancing the cancer cells’ radiosensitivity	[368]
Bromocriptine	Carboxyl or Hydroxyl conjugated multiwalled carbon nanotubes	Dopamine receptor	Carboxyl or hydroxyl group	Carbon nanotubes, thionyl chloride, tetrahydrofuran	A549, QU-DB	26.3–32.6		Sustained Release	Bromocriptine act via dopamine receptor and cause cancer cell apoptosis	[268]
Gold NP	Aluminum (III) phthalocyanine chloride tetra sulfonic acid and anti-CD133 bioconjugated goldNP	Photodynamic effect	Aluminum (III) phthalocyanine chloride tetra sulfonic acid and anti-CD133 antibody	Aluminum (III) phthalocyanine chloride tetra sulfonic acid, anti-CD133 antibody	A549	63.91 nm	−14.7		The bioconjugate enhance the gold NPs’ photothermal activity	[286]
Doxorubicin	Au–Pt NP	Photothermal/photodynamic	cRGD, Au	Gold(III) chloride trihydrate (HAuCl4·3H2O), Pluronic^®^ F-127 (F-127), silver nitrate (AgNO3), ascorbic acid, potassium tetrachloroplatinate(II) (K2PtCl4), methyl thiazolyl tetrazolium (MTT), calcein AM, and PI, thiol poly-(ethylene glycol) succinimidylglutaramide, doxorubicin	MDA-MB231	78.4–85.3	−14.8	Sustained release	Porous Au–Pt NPs loaded with doxorubicin modified with cRGD exhibit better drug release patterns, as well as enhanced anticancer properties	[303]
Cantharidin	Mesoporous titanium peroxide NPs	Photodynamic, increased reactive oxygen species	YSA	Tetrabutyl titanate, (3-aminopropyl) trimethoxysilanetitanium butoxide, hydrogen peroxide, heptanoic acid, ethanol, N-(3-dimethylaminopropyl)-N-ethylcarbodiimide hydrochloride, doxorubicin and N-hydroxysuccinimide. Cantharidin (CTD)	A549	150	−21.77		YSA-modified mesoporous titanium peroxide NPs loaded with cantharidin produced reactive oxygen species and increased photodynamic lung cancer apoptosis	[329]
Doxorubicin	Metal–organic framework	Enhanced the loading drug efficacy up to 5 times without affecting the normal cells	RGD	Diphenyl carbomate, KOH, gamma cyclodextrin, RGD peptide, NHS, EDC, low-molecular-weight heparin	A549	150	−25.6	Sustained release	The loaded drug efficacy enhanced the targeted sites	[85]
Doxorubicin	Quantum dots	Folate receptor	Folic acid, 11-mercaptoundecanoic acid	Sodium dihydrogen phosphate, disodium hydrogen phosphate, sodium chloride, potassium chloride, silver nitrate, indium(III) chloride, zinc stearate, 1-dodecanethiol, sulfur, 1-octadecene, oleylamine, MUA, dimethyl sulfoxide, cysteine, lipoic acid, NHS, EDC, doxorubicin hydrochloride, folic acid	A549	11–19	−15.5 ± 3.5	Sustained release	QD nanocrystals modified with folic acid and 11-mercaptoundecanoic acid showed improved cytotoxicity, genotoxicity, and migration inhibitory activity against A549 lung cancer cells	[355]
Doxorubicin	Quantum dots	Overexpressed glycoprotein CD44	Hyaluronic acid	Dicarboxyyl-terminated poly(ethylene glycol), hyaluronic acid, zinc acetate, magnesium acetate, sodium hydroxide, dimethyl sulfoxide, anhydrous N,N-dimethylformamide, doxorubicin	A549		−0.0521 −1.90	Sustained release	Shows synergistic effect of Zn2+and doxorubicin for antitumor activity	[354]

## 6. Conclusions

Lung cancer has a lower survival rate due to the complexity of delivering the active drugs to the targeted sites. Biological barriers, behavioral nature, and tumor heterogeneity impact the delivery of drugs to lung cancer. There have been many attempts to overcome the barriers through different therapeutic approaches such as chemotherapy, immunity modulation therapy, radiation therapy, chemotherapy, stereotactic body radiotherapy, etc. Recently, other than the above therapeutic options, interest in targeted drug delivery systems has increased as adjuvant therapy in both the early and late stages of disease progression. The reason is that most of the above-mentioned conventional therapies became resistant after a certain period, and the therapeutic accumulation in the intracellular region was enough to cause toxicity in the tumor microenvironment. In addition to that, conventional therapies are unlikely to enter the tumor microenvironment.

In our study of nanocarrier-based targeting drug delivery to overcome the TME barriers, we have found that particle size and active targeting using receptor-based bioconjugating agents play roles in bypassing the TME to enhance the targeting efficacy of the loaded drug.

Different studies on lung cancer found that the inflammatory mediators were overexpressed, especially IL-6. A high-affinity protein can block it. The nanocarrier biofunctionalized with proteins such as RGD can be useful for targeting cancer cells. Further, folic acid deficiency promotes IL-6/JAK-1/pSTAT3 interactions in astrocytes after ischemia-reperfusion. So, folic acid biofunctionalized nanocarriers may be another approach to improving the targeting precision. Further, the PEGylation of NPs reduces the interaction with serum proteins and enhances the stability of the nanocarrier in the reticuloendothelial system.

The selection of the nanocarrier for lung cancer treatment primarily depends upon factors such as the physiochemical nature of the drug, solubility, permeability, molecular weight, and aqueous stability. Secondly, the nanocarrier’s specificity, viz., particle size and surface geometry, biocompatibility, and nano-toxicity are important considerations in designing nanocarriers. Biodegradable nanocarriers are often preferred as they can be metabolized and eliminated from the body, reducing the risk of long-term toxicity.

In this study, we have found multiple nanocarriers with different possibilities. Depending on the requirements and targeting strategy, nanocarriers can be modified to optimize the required outcome.

## 7. Future Prospective

In light of advances in nanotechnology, various research studies are underway to find more convenient cancer treatments. NSCLC remains a substantial clinical challenge, though chemotherapy and surgery are the only standards of care. Drug delivery to the targeted site remains challenging despite newer drugs for different histological subtypes and driver mutations. So, the emphasis on the nanocarrier drug delivery system as an add-on therapy to the current regime will lead to greater effectiveness. According to different studies, we found that biofunctionalized inorganic metal compounds with organic compound complex-loaded drugs may be a carrier system for NSCLC targeted therapy. Especially, with active targeting through surface modifications of receptors overexpressed in lung cancer cells (folic acid, peptide, somatostatin). The biofunctionalization of the nanocarrier enhances biosystem interaction, cellular uptake, immune system escape, and vascular alteration to penetrate the tumor microenvironment. The inorganic metal compounds have a photothermal effect that scavenges reactive oxygen species. Further, the loaded pathway-blocking agents can inhibit rapid cancer cell growth.

A redox-sensitive prodrug and pH-responsive carbon nanotubes can ensure active form in conditions that suit the tumor microenvironment. In addition, modified organic andinorganic NP carrier systems may also be useful in lung cancer-targeted therapies.

In this study, we have discussed the different nanocarrier systems and their TME bypassing strategies. This study will help to develop new targeted therapeutics using a modified bioconjugate hybrid nanocarrier that can act through active targeting by bypassing TME. Further, this study will give an idea of different nanocarrier efficacies in a concise form, along with their mechanisms. It will help to compare nanocarriers in diverse conditions for developing personalized therapy.

## Data Availability

Not applicable.
